# RMS: a ML-based system for ICU respiratory monitoring and resource planning

**DOI:** 10.1038/s41746-025-02081-4

**Published:** 2025-12-19

**Authors:** Matthias Hüser, Xinrui Lyu, Martin Faltys, Alizée Pace, David Berger, Marine Hoche, Stephanie L. Hyland, Hugo Yèche, Manuel Burger, Tobias M. Merz, Gunnar Rätsch

**Affiliations:** 1 Department of Computer Science, ETH Zürich, Zürich, Switzerland; 2 Swiss Institute for Bioinformatics, Lausanne, Switzerland; 3 Swiss Data Science Center, Zürich, Switzerland; 4 Department of Intensive Care Medicine, University Hospital, University of Bern, Bern, Switzerland; 5 Department of Intensive Care, Austin Hospital, Melbourne, VIC Australia; 6 AI Center, ETH Zürich, Zürich, Switzerland; 7 Microsoft Research, Cambridge, UK; 8 Cardiovascular Intensive Care Unit, Auckland City Hospital, Auckland, New Zealand; 9 Medical Informatics Unit, Zürich University Hospital, Zürich, Switzerland; 10 Department of Biology, ETH Zürich, Zürich, Switzerland

**Keywords:** Respiratory signs and symptoms, Information technology, Scientific data

## Abstract

Acute hypoxemic respiratory failure (RF) occurs frequently in critically ill patients and is associated with substantial morbidity, mortality and resource use. We developed a comprehensive machine-learning–based monitoring system to support ICU physicians in managing RF through early detection, continuous monitoring, assessment of extubation readiness, and prediction of extubation failure (EF). In study patients, the model predicted 80% of RF events with 45% precision, identifying 65% of events more than 10 hours before, significantly outperforming standard clinical monitoring based on oxygenation index. The model was successfully validated in an external ICU cohort. We also demonstrated how predicted EF risk could help prevent extubation failure and unnecessarily prolonged ventilation. Lastly, we illustrated how prediction of RF risk, along with ventilator need and extubation readiness, helped ICU resource planning for mechanical ventilation. Our model predicted ICU-level ventilator demand 8–16 hours ahead, with a mean absolute error of 0.4 ventilators per 10 patients.

## Introduction

Acute hypoxemic respiratory failure (RF) is a common occurrence in intensive care unit (ICU) patients and is associated with high morbidity, mortality, and high resource use^1,2^. Hypoxemic RF (Type I RF) is the most common type of respiratory failure^3^ and its severity is defined by the P/F (PaO_2_/F_I_O_2_) ratio, with values below 200 mmHg corresponding to moderate and below 100 mmHg to severe RF. Treating patients with RF involves a sequence of clinical evaluations, including identifying RF and the need for mechanical ventilation, monitoring the recovery of lung function, determining the right time to stop mechanical ventilation, and assessing the risk of complications after tracheal extubation.

For optimal clinical decision-making, it is paramount to continuously monitor the patient’s clinical state in an attempt to predict their future clinical course. ICU physicians base their treatment decisions mostly on intermittent clinical assessments and trend evaluation of monitored vital signs stored in electronic patient data management systems. In the increasingly complex ICU environment, clinicians are confronted with large amounts of data from a multitude of monitoring systems of numerous patients. The quantity of data and the possibility of artifacts increases the risk that clinicians will not readily recognize, interpret, and act upon relevant information, potentially contributing to suboptimal patient outcomes and increased ICU resource expenditure^4^, compared to optimal care. Large datasets involving multiple data points on many patients are ideal for automatic processing by machine learning (ML) algorithms^5,6^. To facilitate such advancements, we previously published the High time Resolution Intensive Care Unit (HiRID) Dataset, which encompasses approximately 34,000 ICU admissions^7^. ML has been used to develop decision support systems for various conditions in the ICU, such as acute respiratory distress syndrome (ARDS)^8–12^, circulatory failure^7^, sepsis^13–15^, and renal failure^16^.

We aimed to develop a comprehensive, ML-based respiratory monitoring system (RMS), consisting of multiple subsystems to simplify and expedite the management of individual patients with RF and to optimize ICU ventilator resource planning. For individual patients, the system predicts the risk of hypoxemic RF (RMS-RF) and the need for mechanical ventilation (RMS-MV_Start_), continuously monitors changes and improvements of the respiratory state, and predicts the remaining time of required mechanical ventilation (RMS-MV_End_) and probability of successful extubation (RMS-EF). We investigated how using respiratory state predictions on a patient-by-patient basis could enable estimating the future number of patients in need of mechanical ventilation on a shift-to-shift short-term basis (resource planning). In addition, we prepared a new version of the dataset, HiRID-II, which we anticipate will significantly expand both the number of included patients and the range of available clinical variables.

We hypothesized that our ML system could predict the relevant respiratory events throughout the treatment process of individual patients accurately and early, both in the development dataset and when validated in externally sourced data. In addition, we intended to develop a resource management support tool to predict ICU-level future mechanical ventilator use by integrating all RMS scores across ICU patients.

## Results

### Preparation of an extended HiRID dataset (HiRID-II)

We present the High Time Resolution Intensive Care Unit Dataset II (HiRID-II), a substantial update to HiRID-I^17^, that will be made available to the research community on Physionet.org^18,19^. This new dataset contains 60% more ICU admissions than its predecessor (Table 1 and Supplementary Fig. 1a). Additionally, the number of variables has increased from 209 to 310 (Supplementary Fig. 1b). The dataset was k-anonymized in regard to age, weight, height, and gender, reducing the number of admissions from 60,503 to 55,858^20^. Admission dates were randomly shifted to further reduce the risk of identification of individual patients. To allow the assessment of model generalization to the future, the dataset was divided into temporal splits while respecting k-anonymization (Supplementary Fig. 1c). A high-resolution external evaluation dataset, extracted from the AmsterdamUMCdb^21^, was used to test the generalizability of RMS to other health care systems (Supplementary Fig. 1d). Preliminary analysis of the HiRID-II dataset revealed strong correlations between the occurrence of RF and EF with ICU mortality, confirming prior results^1^ and motivating our proposed RMS (Supplementary Fig. 2).

**Table 1:** Characteristics of the HiRID-I and HiRID-II datasets

	HIRID-I	HIRID-II
Data set size		
Year of admission	2008–2016	2008–2019
No. of patients	33,905	55,858
No. of meta-variables	209	310
No. of variables	710	890
No. of patient-years	356.73	552.77
Demographics		
Gender	Male: 63.5%, Female: 36.5%	Male: 63.1%, Female: 36.9%
Age [years]	66.0 [54.0, 75.0]	66.0 [54.0, 75.0]
Diagnostic group		
Neurologic/Neurologic surgery	17.4%/11.3%	20.1%/13.8%
Cardiovascular/Cardiovascular surgery	13.0%/23.7%	10.9%/20.0%
Gastrointestinal/Gastrointestinal surgery	5.0%/5.4%	4.7%/5.6%
Respiratory/Respiratory surgery	7.5%/1.9%	6.9%/1.6%
Trauma/Trauma surgery	4.5%/0.7%	5.4%/1.0%
Other	9.6%	9.9%
Outcomes		
ICU Mortality	6.1%	5.5%

Age is reported as median and interquartile range (IQR). The statistics were computed on the HiRID datasets after k-anonymization. HiRID-II includes patients from HiRID-I.

### Development of a continuous monitoring system for respiratory management

**Fig. 1:**
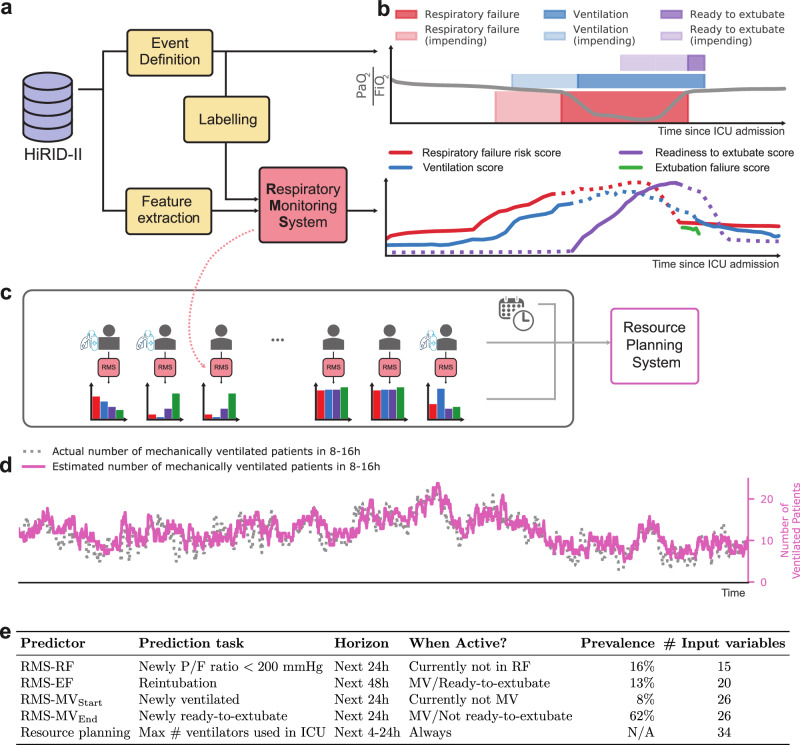
Overview of the RMS decision support system for respiratory state management, and its extension for ICU-level ventilator resource planning. **a** Flow diagram for the development of RMS predictors at the individual patient level. Time series were extracted from the HiRID-II dataset and gridded to a 5-min resolution, and features were computed. Respiratory failure/ventilation/ready-to-extubate periods were annotated and machine learning labels created. **b** The respiratory monitoring system consists of four scores which are active during different time periods of the ICU stay, according to the current respiratory and ventilation state of the patient. **c** Flow diagram for the development of a resource monitoring system at the ICU level. For all current patients in the ICU, the four scores were integrated to predict the probability that a patient will require mechanical ventilation within a future time horizon. **d** Example time period of 3 months, displaying the actual number of ventilated patients and the predicted number as estimated by RMS in the next 8–16 h. **e** Overview of prediction tasks solved by RMS for individual patients (RMS-RF/RMS-EF/RMS-MV_Start_/RMS-MV_End_) as well as at the ICU level. For RF, MV_Start_ and MV_End_ we provide the event prevalences in the test set at times when the patient is stable, not ventilated, or ventilated, respectively.

We developed a comprehensive ML-based RMS composed of four interrelated predictive models that, together, cover the respiratory trajectory of patients (Fig. 1a). RMS-RF estimates every 5 min the risk of moderate to severe hypoxemic respiratory failure (P/F ratio <200 mmHg) in the next 24 h. RMS-MV_Start_ predicts the need for mechanical ventilation, while RMS-MV_End_ determines if the patients will be ready to be liberated from ventilatory support; both tasks forecast risk within the subsequent 24 h. Finally, RMS-EF evaluates the likelihood of successful extubation at given time points, when the patient already meets formal criteria.

For each time point, we determined if a patient is currently in moderate or severe hypoxemic RF (P/F ratio <200 mmHg), mechanically ventilated, or ready to be extubated. Readiness to extubate (REXT) status at each time point was defined using a heuristic scoring system determined by gas exchange, respiratory mechanics, hemodynamics, and neurological status. A score threshold was manually selected after inspection of the time series by an experienced ICU clinician (Fig. 1b). At these time points, the patient could be extubated according to formal criteria, but extubation failure can still occur. The current ventilation status was derived from the presence of ventilator-specific parameters.

Positive labels for future RF were defined as time points when the patient was not currently in RF but would exhibit RF in the next 24 h (“impending RF”); while negative labels were assigned to time points when the patient was not currently in RF and would remain stable during the next 24 h. For every extubation event, we determined whether it failed (reintubation necessary within 48 h after extubation) and used it as the label for extubation failure. Labels for ventilation onset and readiness to extubate prediction were positive at time points when the patient was currently not ventilated/ready-to-extubate but would be in the next 24 h (Fig. 1b). In HiRID-II, 43.7% and 46.2% of all patients had RF events and required mechanical ventilation, respectively. The dataset contained 23,861 extubation events, of which 11.1% extubations failed.

To measure PaO_2_, an arterial blood sample is necessary. PaO_2_ is therefore only periodically available at a low temporal resolution. For continuous high-resolution hypoxemic RF labeling, a continuous estimation of the current PaO_2_ is required. An underlying physiological principle determines the binding and dissociation of oxygen and hemoglobin, yielding a sigmoid correlation between arterial oxygen saturation (SpO_2_) and PaO_2_^22–24^. A model based on the continuously available SpO_2_ can therefore estimate continuous PaO_2_. We developed an ML algorithm to produce continuous estimates of PaO_2_ based on SpO_2_ and other relevant variables determining the hemoglobin-oxygen dissociation curve. The algorithm outperformed the existing nonlinear Severinghaus-Ellis baseline^25,26^ for estimating PaO_2_ values from non-invasive SpO_2_ measurements (Supplementary Fig. 3). By integrating PaO_2_ estimates with continuously available F_I_O_2_ data, we derived P/F ratio estimates on a 5-min time grid.

The RMS produces four individual scores active at different stages of the RF management process. All four subsystems are based on manual feature engineering and LightGBM^27^ predictors, similar to our previous work^7^. Prior analyses on HiRID-I for circulatory failure and a related respiratory failure task have shown superior performance of LightGBM compared to other models including deep learning^7,28^. The predictor for RF (RMS-RF) used 15 clinical variables (Supplementary Data 3). As in Hyland et al.^7^, the system triggered an alarm if the RF score exceeded a specified threshold. It was silenced for 4 h afterwards. The alarm system was reset when the patient recovered from an event and could reactivate 30 min later. The extubation failure (RMS-EF) predictor used 20 clinical variables (Supplementary Data 3). The RMS-RF & RMS-EF variable sets were identified separately for the two tasks, using greedy forward selection on the validation set of five data splits. The models for ventilator use (RMS-MV_Start_) and extubation readiness (RMS-MV_End_) used the union of the parameters of the two main tasks, consisting of 26 variables in total (Supplementary Data 3).

We utilized the four risk scores to predict short-term mechanical ventilator resource requirements by training a meta-model (Fig. 1c). The resource planning problem was divided into two sub-problems; predicting the future ventilator use for already admitted ICU patients, and predicting the requirement for mechanical ventilation for newly admitted non-elective patients in the near future. We excluded elective patient admissions as their resource use is typically known well in advance (hence, no prediction is needed). The predictor uses date and time information as well as summary statistics regarding ventilator use and patient numbers derived from the ICU dataset. A LightGBM^27^ regressor was trained to solve both sub-problems. For admitted ICU patients, it predicts the necessity for mechanical ventilation in the short-term future, as well as the total number of ventilators required for all admitted patients as an aggregate of the individual predictions (Fig. 1d).

All elements of the developed system (Fig. 1e), including data preprocessing, annotation, prediction task labeling, and both training and prediction pipelines, are made available under an open source license facilitating the reproducibility and reuse of the methodology and results.

### RMS-RF predicted RF early with high precision and reduced false alarms compared to clinical baselines

We developed a model that continuously evaluates the likelihood of a patient developing hypoxemic RF within the next 24 h, updating its predictions every 5 min throughout the ICU stay. We define RF as a moderate or severe reduction in oxygenation, reflected by a P/F ratio below 200 mmHg. To focus on impending deterioration, the model only generates predictions when the patient is not currently experiencing RF. Conversely, if the patient remains stable and does not meet criteria for RF over the subsequent 24 h, the model recognizes a low-risk state. The early prediction of hypoxemic RF is crucial for timely intervention and may reduce the number of unfavorable patient outcomes and improve overall healthcare quality. By accurately forecasting these events, RMS-RF may not only improve clinical decision-making but also allow physicians to commence treatment early, thereby mitigating the risk of more severe respiratory complications.

**Fig. 2:**
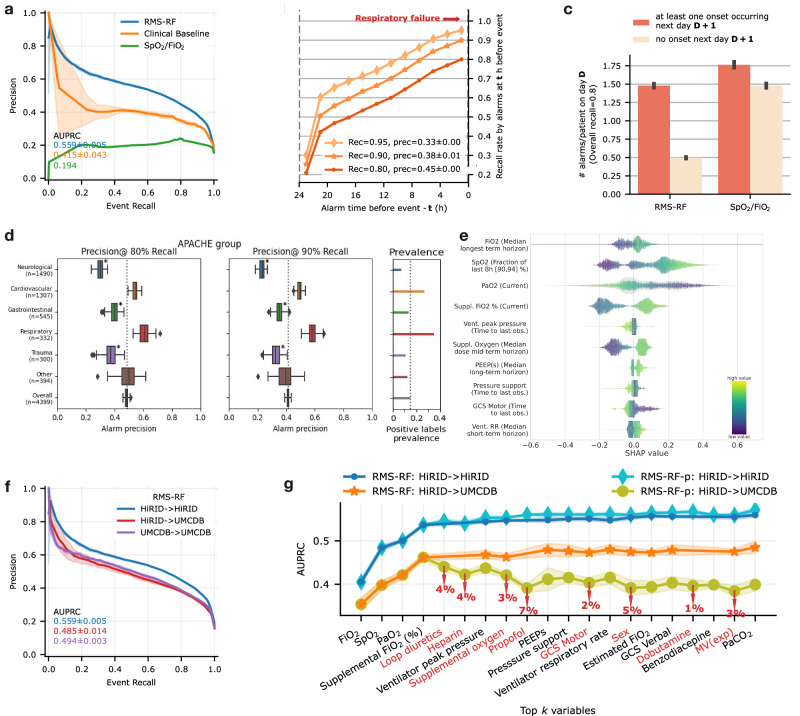
RMS-RF: model performance/feature inspection of the respiratory failure prediction model. **a** Model performance of RMS-RF compared with a decision-tree-based clinical baseline and a threshold-based alarm system based on the current SpO_2_/F_I_O_2_ ratio. **b** The RMS-RF system’s performance was evaluated in terms of earliness of its alarms. Specifically, this is measured as the proportion of respiratory failure events for which it provides early warning during a fixed prior time period and considering only events with a prior sufficient stability period. **c** Comparison of generated false/true alarm counts of RMS-RF compared with a fixed threshold alarm system, both for patients with events, and patients without events on a given day. **d** Alarm precision at event recalls of 80% and 90% of the RMS-RF model by admission diagnostic group category. The model was re-calibrated for each sub-group using information available at admission time to achieve a comparable event recall. **e** Feature inspection using SHAP values for the most important features for predicting respiratory failure, depicting the relationship between feature values and SHAP values. **f** External validation of RMS-RF in the AmsterdamUMCdb dataset^21^. Internal, transfer as well as retrain performance in UMCdb is displayed. **g** RMS-RF performance changes as the most important variables are added incrementally to the model, for the internal HiRID setting, and the transfer setting to UMCdb. Model transfer issues between the two hospital centers existed if medication variables were included in RMS-RF, denoted as the RMS-RF-p model variant. Markers denote the variables included in the models, and red colors denote variables which decrease performance when added to the model in the transfer setting.

The RMS-RF model achieved an area under the alarm/event precision recall curve^7^ (AUPRC) of 0.559 with an alarm precision of 45% at an event recall of 80%. The model had an area under the receiver operating characteristic curve (AUROC) of 0.839 (Supplementary Fig. 4a). We observed that RMS-RF significantly outperformed two comparator baselines, a decision tree that uses the current value of respiratory and other parameters (SpO_2_, F_I_O_2_, PaO_2,_ Positive end-expiratory pressure (PEEP), RR, Ventilator presence, HR, GCS) as well as a clinical threshold-based system based on the SpO_2_/F_I_O_2_ ratio (Fig. 2a). RMS-RF was well calibrated, in contrast to the two baselines (Supplementary Fig. 4b). The system detected 65% and 78% of respiratory failure events at least 10 h before they occurred when set to an event recall of 80% and 90%, respectively (Fig. 2b). Compared to the SpO_2_/FIO_2_ threshold, our system generated two-thirds fewer false alarms per day on days without respiratory failure (Fig. 2c). The model performance increased with inclusion of data up to 25% of the total dataset size, while no further improvements were observed when using more data (Supplementary Fig. 4c). Model performance was highest in patients across cardiovascular and respiratory diagnostic groups (alarm precisions 55% and 60% at 80% event recall, respectively). Lower performance was observed in neurologic and trauma patients (Fig. 2d). Performance varied in groups determined by age and gender^29^ (Supplementary Fig. 4d, e), and slightly declined for the most severely ill patient group in terms of APACHE score (Supplementary Table 2). RMS-RF exhibited physiologically plausible relationships between risk and clinical variables, according to SHapley Additive exPlanations (SHAP)^30^ values (Fig. 2e and Supplementary Fig. 5). A sensitivity analysis investigating different P/F ratio thresholds and PaO2 estimation models (incl. SpO_2_/F_I_O_2_ ratio) showed similar performance (Supplementary Tables 3–5).

The proposed RMS-RF model used a small number of physiological parameters and ventilator settings. Slightly diminished performance was observed when the HiRID-II-based model was externally validated in the AmsterdamUMCdb dataset^21^. There was no significant performance improvement observed through retraining with local data (Fig. 2f; both have 38% alarm precision at 80% event recall). We excluded medication variables to reduce the effect of differences in medication policies in different hospitals. A variant of RMS-RF including medication variables (RMS-RF-p) achieved only minor gains in internal HiRID performance (Fig. 2g) and exhibited poor transfer performance to UMCdb (Supplementary Fig. 6a). Medication policy differences between HiRID-II and UMCdb were analyzed to investigate these drops in transfer performance, indicating that these were generated by differences in the use of loop diuretics, heparin, and propofol (Fig. 2g and Supplementary Fig. 6b, c).

### RMS-EF predicted extubation failure with high precision and was well-calibrated

**Fig. 3:**
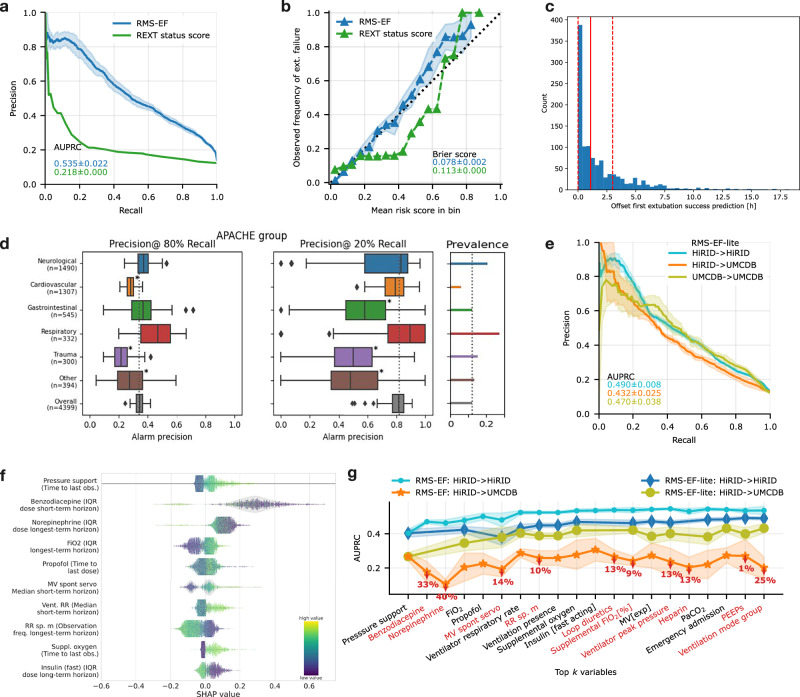
RMS-EF: model performance analysis and feature inspection. **a** Model performance compared with a baseline based on clinically established criteria for readiness to extubate in terms of recall/precision. **b** Risk calibration of the score for predicting extubation failure at the time of extubation, compared with the baseline. **c** Distribution of time span between the earliest extubation success prediction of RMS-EF prior to the time point of successful extubation, for correctly predicted successful extubations. The earliest time is defined as the first time point from which RMS-EF continuously predicts “extubation success” while the patient is ready-to-extubate. Red dashed lines denote the 25, 75 percentiles, and the red solid line denotes the median, respectively. **d** Performance stratified by admission diagnostic group in terms of precision at 80% and 20% recall. The model was re-calibrated for each sub-group using information available at admission time, to achieve a comparable recall. A * next to a bar indicates significantly different from average performance. **e** Performance of the RMS-EF-lite model, which is obtained by excluding medication variables from RMS-EF, when trained/tested on the HiRID-II dataset, transferred to the UMCdb dataset, and retrained in the UMCdb dataset. **f** Summary of SHAP value vs. variable distribution for the most important feature of each of the top 10 important variables contained in the RMS-EF model. **g** Performance of the RMS-EF and RMS-EF-lite models in the internal and transfer settings as variables are added incrementally to the model ordered by performance contribution (greedy forward selection performance on the validation set). Red marked percentages on the orange curve denote relative performance loss in the transfer, when adding the variable to the model. Variables are in red font if their inclusions lead to performance loss in the transfer setting.

The accurate prediction of extubation failure is a critical aspect of patient management in intensive care, enabling clinicians to make informed decisions about the ideal timing of extubation. By utilizing RMS-EF to predict the risk of extubation failure, physicians could judiciously determine whether to proceed with or delay extubation based on a quantifiable risk threshold, potentially reducing the likelihood of complications associated with both premature extubation or unnecessary prolongation of mechanical ventilation. We compared the developed RMS-EF predictor to a threshold-based scoring system, which counts the number of violations of clinically established criteria for readiness to extubate at the time point when the prediction is made (REXT status score). RMS-EF significantly outperformed the baseline (Fig. 3a) with an AUPRC of 0.535 and an AUROC of 0.865 (Supplementary Fig. 7a). We also analyzed calibration and observed a high concordance between observed risk of extubation failure and RMS-EF with a Brier score of 0.078; in contrast to the baseline (Fig. 3b). The precision for predicting extubation failure was 80% at a recall of 20% indicating that RMS-EF can effectively identify the patients at highest risk. RMS-EF predicted successful extubation at least 3 h prior to the time point when extubation effectively takes place in 25% of events (Fig. 3c). As with RMS-RF, no major improvements in model performance were observed when using more than 25% of the training data (Supplementary Fig. 7b). Performance across diagnostic groups was similar, with RMS-EF performing best in respiratory patients (Fig. 3d). We observed that the performance in female patients and older age groups was slightly inferior (Supplementary Fig. 7c, d). As for RMS-RF, performance was slightly reduced for the most severely ill patient group (Supplementary Table 2). As RMS-EF is based almost exclusively on variables that are influenced by clinical policies (Fig. 3f and Supplementary Fig. 8), which likely differ in different hospitals, it transferred poorly to the UMCdb dataset^21^ (Supplementary Fig. 7e). However, a variant of our model can be constructed without medication variables, which transferred well to the UMCdb dataset with slightly reduced internal performance (Fig. 3e; AUPRC 53.5% vs. 49% for HiRID). Accordingly, the analysis of medication policies revealed major differences for ready-to-extubate patients between HiRID-II and UMCdb (Supplementary Fig. 7f/g). SHAP value analysis^31^ showed that the RMS-EF risk score was dependent on several parameters determined by treatment policies (Fig. 3f and Supplementary Fig. 8). Severe loss of transfer performance resulted from the inclusion of sedatives and vasopressors in the model (Fig. 3g).

### Predicting intubation and readiness-to-extubate for individual patients

We evaluated prediction of ventilation onset (RMS-MV_Start_) and readiness to extubate (RMS-MV_End_) within the next 24 h also on a patient level. We observed high discriminative performance with AUROCs of 0.914 and 0.809 (Supplementary Fig. 9a, b), event-based AUPRCs of 0.528 and 0.910 (Supplementary Fig. 9c, d), for RMS-MV_Start_ and RMS-MV_End_, respectively, and the models were well calibrated (Supplementary Fig. 9e, f).

### Integrating all RMS scores of individual patients for planning ICU-level resource allocation

**Fig. 4:**
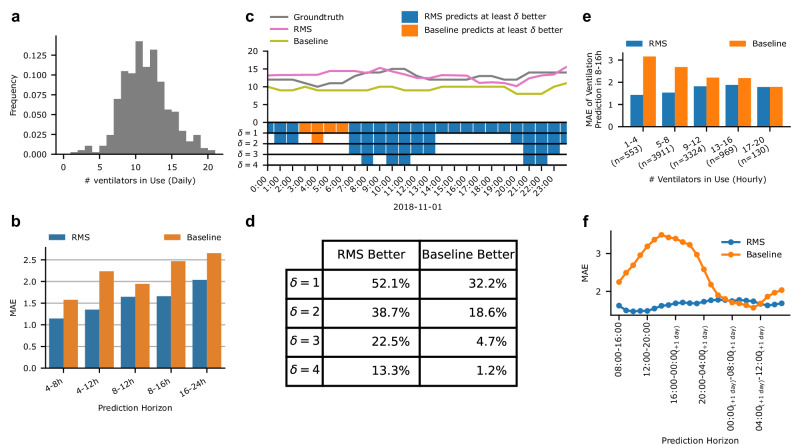
Model performance of the integrated system for ventilator resource planning (RMS) in an ICU with an effective capacity of 42 beds. **a** Observed ventilator usage pattern in the HiRID-II dataset, in terms of days on which a particular number of ventilators is used. **b** Performance of RMS compared with the baseline, in terms of mean absolute error (MAE) for predicting the maximum number of used ventilators at a fixed horizon in the future. **c** Example of a typical ICU setting shown for a duration of one day, annotated with ground-truth, RMS, and baseline predictions. In the rug plot, relevant better predictions are marked for the offsets 1–4. **d** RMS compared with the baseline, for different absolute differences of predictions (1–4) in the rows, for a prediction horizon of 8–16 h in the future. The table entries denote the proportion of time points in which either RMS or the baseline is better by at least the difference of the row. **e** Performance of RMS for different current ventilator ICU usage scenarios, ranging from low usage, to high usage, compared with the baseline, for a prediction horizon of 8–16 h in the future. The number of time points falling into each bin is denoted in parentheses. **f** Performance of RMS, by hour of the day when the prediction is performed, compared with the baseline, for a prediction horizon of 8–16 h in the future.

Using the predictions for the four models focusing on respiratory failure (RMS-RF), extubation failure (RMS-EF), ventilation onset (RMS-MV_Start_), and readiness to extubate (RMS-MV_End_), we developed a combined model predicting the number of ventilators in use for non-elective patients at a specific future horizon. Preliminary analysis of the HiRID-II dataset demonstrated substantial variation in demand for ventilators each day, underscoring the need for a model to aid resource planning (Fig. 4a).

We trained a meta-model using the four scores to predict the number of ventilators in use in the ICU at future time horizons every hour (4–8 h, 4–12 h, 8–12 h, 8–16 h, 16–24 h; Fig. 4b). We compared it with a baseline that predicts that the number of non-elective patients requiring mechanical ventilation remains stable. We observed that the proposed model clearly outperforms this baseline in terms of mean absolute error (MAE), with the largest relative gain in longer prediction horizons (Fig. 4b). In 39% of time points the model’s predictions were at least two ventilators closer to ground-truth, for predicted ventilator use in 8–16 h (Fig. 4c, d). RMS outperformed the baseline for the majority of ICU ventilator utilization scenarios (Fig. 4e), with the largest improvement over the baseline when the respirator use is below the maximum capacity (Fig. 4e) and for predictions of ventilator use during day hours (Fig. 4f).

### Explorative joint analysis of RMS scores throughout the ICU stay

**Fig. 5:**
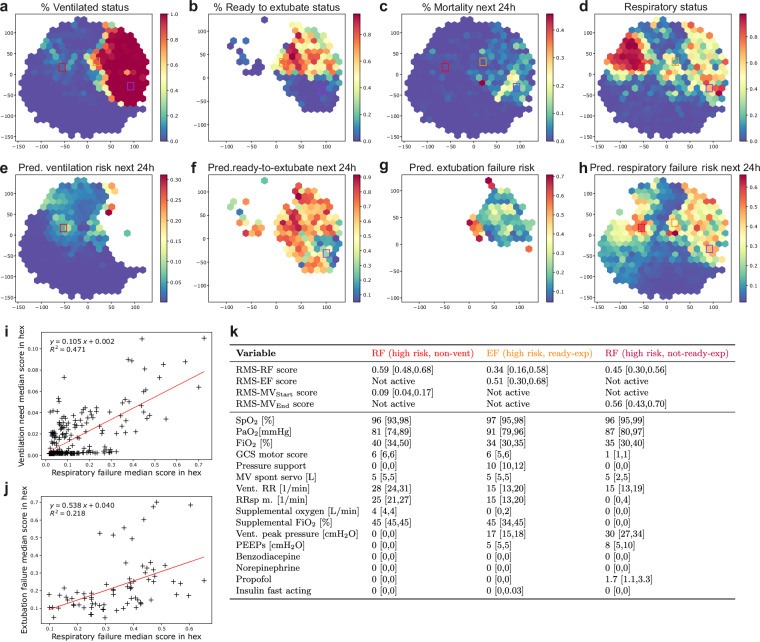
Joint analysis of the four scores produced by RMS overlaid on a t-SNE embedding based on important respiratory parameters. In all plots, *x*, *y* axes represent the two dimensions of the two-dimensional t-SNE embedding space. **a** Hexes are colored by the proportion of time points in the hex for which the patient is ventilated. **b** Hexes are colored by the proportion of time points in the hex for which the patient is ready-to-extubate, given the patient is ventilated. **c** Hexes are colored by observed 24 h mortality risk. **d** Hexes are colored by the proportion of time points in the hex for which the patient is in respiratory failure. **e**–**h** The color of the hex denotes the median RMS-MV_Start_/RMS-MV_End_/RMS-EF/RMS-RF scores of the time points assigned to the hex, respectively. **i** Relationship of median respiratory failure score (RMS-RF) and median ventilation need score (RMS-MV_Start_) in hexes for time points where both scores are active. The *p*-value of a Wald test for a non-zero regression line slope is 1.5 × 10^−36^. **j** Relationship of median respiratory failure score (RMS-RF) and median extubation failure score (RMS-EF) in hexes for time points where both scores are active. The *p*-value of a Wald test for a non-zero regression line slope is 2.7 × 10^−5^. **k** Score and input value distribution of time points assigned to three selected hexes for the 16 variables used as input for the t-SNE. The median is reported, and numbers in square brackets refer to the interquartile range.

We analyzed the relationship of the four RMS scores produced at each time point of the ICU stay by embedding the most important parameters for respiratory failure and extubation failure prediction (union of the top 10 variables identified for each task, current value feature) using t-distributed stochastic neighbor embedding (t-SNE^32^) with subsequent discretization into hexes. This approach produces a two-dimensional hex-map that defines subsets of comparable patient states that can be compared across different characteristics, i.e., between the panels for the hex. We observed that the space is divided into two distinct states, corresponding to time points when the patient is ventilated or not ventilated (Fig. 5a). The region of ventilated patients is further subdivided, with patients in the upper part being more likely to be ready-to-extubate (Fig. 5b). As expected, the ventilated and not ready-to-extubate region has the highest observed 24 h mortality (Fig. 5c). Patients experiencing respiratory failure were concentrated in a compact region in the area corresponding to non-ventilated patients, as well as scattered throughout the area corresponding to ventilated patients (Fig. 5d). States with high risk of future ventilation need according to RMS-MV_Start_ are close to the boundary of the ventilated region (Fig. 5e). Readiness to extubate scores show a less clear pattern, but scores tend to be higher in the upper part of the ventilated region, which is also enriched in states in which patients are ready-to-extubate (Fig. 5f). For RMS-EF, high scores are concentrated in two distinct regions at the edge of the ventilated region (Fig. 5g). Lastly, RMS-RF scores are high close to the boundary of patients already in respiratory failure (Fig. 5h). The median risk scores of hexes for respiratory failure/ventilation need are strongly positively correlated with an *R*^2^ of 0.471 (Fig. 5i). Likewise, respiratory failure and extubation failure scores were moderately positively correlated (Fig. 5j). For RMS-EF/RMS-MV_End_ scores, no correlation could be observed (Supplementary Fig. 10). For three exemplary hexes with predominantly (1) non-ventilated patients but high RMS-RF score, (2) ready-to-extubate patients but high RMS-EF score, and (3) not-ready-to-extubate patients but high RMS-RF score, the distribution of clinical parameters was analyzed, showing plausible relationships with clinical parameters. For instance, the non-ventilated patients with the highest RF risks had low PaO_2_, high (supplemental) F_I_O_2_ and high respiratory rates (Fig. 5k).

## Discussion

We presented an ML-based system for the comprehensive monitoring of the respiratory state of ICU patients (RMS). RMS consists of four highly accurate scoring models that predict the occurrence of respiratory failure, start of mechanical ventilation, readiness to extubate, as well as tracheal extubation failure. By combining the prediction scores of all admitted patients at any time point and by accounting for the likelihood of future admissions, RMS facilitated the accurate prediction of the near-future cumulative number of patients requiring mechanical ventilation, which may help to optimize resource allocation within ICUs.

The HiRID-II dataset released alongside this work is a rich resource for broad-scale analyses of ICU patient data. It represents an important advance over HiRID-I, both in terms of the number of included patients and the number of clinical parameters that are included. Our initial analysis of the HiRID-II dataset identified clinically significant links; both the presence and duration of respiratory failure, as well as extubation failure, were associated with increased ICU mortality, indicating distinct yet interconnected risk factors. These insights highlight the critical need for advanced alarm systems for clinical settings to reduce the risks associated with respiratory and extubation failure. The release of the HiRID-II dataset on Physionet^18,19^ will offer numerous opportunities for further research, allowing for more in-depth investigations into various aspects of ICU patient care and outcomes.

RMS-RF predicts respiratory failure throughout the ICU stay, and alarms for impending failure were typically triggered at least 10 h before the event. This early warning has the potential for clinicians to optimize medical therapy and potentially prevent the need for intubation. The transparent break-up of the model’s alarm output into SHAP values of the most relevant parameters may inform clinician understanding and guide their actions. RMS-RF outperformed a baseline representing standard clinical decision-making based on SpO_2_ and F_I_O_2_, and significantly reduced the number of false alarms. It produces RF-specific alarms and silences them within a specified period of time after the model triggers an alarm, reducing alarm fatigue, which is a major issue for ventilator alarms^33^. Prior to respiratory failure, only 1.5 alarms per patient/day were raised, which is manageable for the clinical personnel and unlikely to cause alarm fatigue. Reassuringly, only variables directly associated with respiratory physiology or ventilator settings were found to be predictive of impending respiratory failure. RMS-RF demonstrated its highest precision in individuals admitted with cardiovascular or respiratory admission diagnoses, while its performance notably declined in neurologic patients. In these patients ventilatory management is often determined by the need to protect a compromised airway in patients with altered levels of consciousness and not by the presence of RF per se^34^. Performance for specific comorbidity types, such as COPD or ARDS, could not be assessed due to the lack of diagnoses besides the admission category in our dataset.

To date, few externally validated ML models continuously predicting acute respiratory failure in the ICU have been reported. Recent works by Le et al.^11^, Zeiberg et al.^35^, and Singhal et al.^36^ focus on mild respiratory failure (P/F index <300 mmHg). Other models predict respiratory failure at the time of ICU admission or are only valid for specific cohorts^37–39^.

RMS-EF predicts tracheal extubation failure and significantly outperforms a clinical baseline derived from common clinical criteria for assessment of readiness to extubate status. The model was well calibrated, with almost ideal concordance of the prediction score and observed risk of extubation failure. A potential use case would be to assess the predicted failure risk to determine whether to accelerate or delay the extubation of the patient. At 80% recall, a quarter of correctly predicted extubation successes were recommended more than 3 h before the actual extubation. This exceeds the recall of routinely used readiness tests and suggests that our model could help clinicians to extubate patients earlier. However, in our analysis, we could not ascertain whether a patient was not extubated for reasons not apparent from the data, such as the availability of staff. For clinical use, the model could also be operated at 20% recall with very high precision (80%), to identify patients with a high likelihood that extubation will be unsuccessful. This could caution clinicians from prematurely extubating high-risk patients. For the prediction of extubation failure, various models have been proposed^40–45^. The largest cohorts to date were used in the works by Zhao et al.^45^, who only validated the model in a cardiac ICU cohort, which limits the generalizability of the results, and Chen et al.^46^, who restricted the evaluation to ROC-based metrics, and do not discuss the clinical implications of the model’s performance.

ML has previously been used to develop support systems for the management of RF patients in the ICU. These included models for detection of ARDS^8–12^ and COVID-19 pneumonitis patients^36,47^, prediction of readiness-to-extubate^48–50^, need for mechanical ventilation^51,52^, and detection of patient-ventilator asynchrony^53^. Existing work focused on single aspects of RF management, often in specific patient cohorts only. Our approach aims to comprehensively monitor the respiratory state throughout the RF treatment process by integrating relevant respiratory-system-related tasks and allowing for joint analysis of risk scores and trajectories. We believe a single and universally applicable system is much more likely to be successfully implemented than multiple fragmented models relating to specific disease entities. A further distinguishing feature of RMS is the 5-min time resolution at which predictions are made, enabling longitudinal analysis of risk trajectories. The dynamic prediction, which is a central feature of our model, is more flexible than traditional severity scores, which are evaluated at fixed time points, such as at 24 h after ICU admission^54^, mainly to predict ICU mortality^55^.

For successful external validation of RMS-RF, it was key to exclude medication variables from the model, as their inclusion was detrimental to model transferability. We hypothesize that this difficulty is caused by the observed medication policy differences between centers. Interestingly, ventilator settings, while also policy-dependent, did not compromise transfer performance in the same way. Investigating and quantifying the underlying policy differences, which made transfer difficult, needs additional research. Model transferability is an important topic in robust ML algorithms for ICU settings, where it has been recently studied in risk prediction in sepsis^15,56,57^ and mortality^58^. Our results suggest that medication variables require special attention to enable transfer. In contrast to RMS-RF, we suggest RMS-EF to be retrained and fine-tuned using the data from the center where it should be applied. The policy differences between different centers proved more detrimental to their performance than for RMS-RF. To ensure the continued clinical relevance and performance of the model, we recommend that it be periodically retrained using local and updated data to reflect evolving medical practices, including post-COVID-19 management, as well as local variances in clinical practices and patient cohorts. The dataset used for model development contains a high proportion of neurological patients, reflective of the patient population in our multidisciplinary tertiary care ICU with neurosurgical services. This may influence the external validity of the system in regions with different patient demographics, though the model has demonstrated robust performance in patients with a respiratory admission diagnosis.

Clinical prediction models for individual patients have been extensively studied. Resource planning in the ICU has received little attention in the ML literature, but came into renewed focus due to the COVID-19 crisis^59^. The first ML-based models to predict ICU occupancy were proposed during the pandemic. Lorenzen et al^59^. predicted daily ventilator use as well as, more generally, hospitalization up to 15 days into the future^60^. The RMS presented here clearly outperformed a baseline method for predicting future ventilator use at the ICU level, which is directly related to staffing requirements^61^. With a MAE of 0.39 ventilators per 10 ICU patient beds used during the next shift (8–16 h), the model is sufficiently precise for practical purposes. Since resource allocation in the ICU depends on local policies and procedures, such a system likely needs to be retrained for every clinical facility for reliable predictions. External validation was not feasible as all public ICU datasets have random date offsets and therefore no information on concurrent patients in the ICU^62^. While the number of mechanically ventilated patients is directly correlated with staffing requirements, other factors—such as the possibly increased workload at the boundaries of mechanical ventilation—may further influence ICU resource allocation, an aspect not addressed in this work.

In this study, we developed predictors of key aspects of respiratory state management, including RF, extubation failure, the need for mechanical ventilation, and readiness for tracheal extubation. These predictors collectively describe various aspects of a patient’s respiratory status in the ICU, which can be used for exploratory analysis. The joint analysis and visualization of risk scores alongside other vital clinical variables yielded discernible clusters that correspond to specific patient states, indicating the potential for risk stratification within a patient population. We observed a separation of patient states into two main clusters that align with ventilated and non-ventilated states, with substructures within these clusters. The patients with highest 24 h mortality risk identified on the hex-map often had depressed levels of consciousness, were more likely to require mandatory modes of mechanical ventilation, had higher peak airway pressure, and required higher PEEP; all indicators of more severe underlying lung pathology. We also identified a cluster of patients who are clinically ready to be extubated and have a low risk of RF but a very high extubation failure risk. These patients required relatively higher airway ventilation pressure and had a low respiratory rate, which are all established risk factors for extubation failure.

The hex-map visualization allows for the monitoring of individual patient states over time with updates, akin to those seen in methodologies like T-DPSOM^63,64^. This dynamic tracking is based on the automated integration of multiple respiratory state dimensions and uses nonlinear dimensionality reduction to provide the position of an individual patient on the map of respiratory health states. We expect that hex-map visualization has the potential to assist clinicians in identifying changes in patients’ clinical states, although the practical implications of this feature require further validation. This represents a different approach to previous work, that mainly tries to understand biological phenotypes of ARDS patients^65–68^ or longitudinal sub-phenotypes of a more specific patient set, like COVID-19 patients^69,70^. Overall, while the hex-map visualization provides an interesting perspective for monitoring of respiratory state in the ICU and can serve as a tool for a more detailed exploration, the presented analysis is exploratory only. Further research is needed to substantiate the clinical relevance of the identified clusters and to explore how this system might integrate into the decision-making processes within the ICU.

Our study tried to avoid certain limitations of retrospective model development studies. Unlike typical single-center studies, our research utilized data from two distinct centers, one for development and another for validation. This approach reduced the risk of overfitting models to a local patient cohort, although it is important to note that external applicability may still vary and retraining on local data will be needed for parts of the proposed RMS. We have incorporated improvements based on our previous work into our models. Unlike earlier systems that were heavily reliant on sporadic clinical measurements, such as serum lactate concentration^7^, our current model uses continuous SpO_2_ monitoring and ventilator data. Use of automated continuous data reduces the influence of clinician-driven decisions on our alarm systems, ensuring a more objective assessment of the patient’s condition. However, the retrospective nature of our data collection is still a limitation. Missing data was partially imputed for respiratory failure annotation, and while this aids in model development, it introduces potential biases. This study does not address how integrating the system into everyday clinical practice might influence treatment or monitoring strategies (a phenomenon known as domain shift^71^). Specifically, if the model would rely heavily on clinician-driven interventions (such as changes in PEEP or the administration of diuretics) as predictors of respiratory failure, any future alterations in clinician behavior (possibly driven by the model implementation) could reduce the model’s predictive accuracy. We constructed clinical baselines as best-effort reference points for comparison, derived from the data available in our cohort. As such, they are not established standards and may miss important clinical elements, such as respiratory effort, imaging, and other contextual factors. Lastly, our assessment of the extubation failure risk score was limited to scenarios of actual extubation events. While we think that the accuracy of this score would be similar in patients nearing readiness for extubation, this cannot be definitively concluded from our retrospective data. Future prospective implementation studies are needed to fully understand the implications of our model in a live clinical setting on patient-centered outcomes. Such studies would likely need local fine tuning of the models and a validated real-time implementation of the ML algorithms in the local EHR. This should include a risk-score display, a clinician notification system, and a defined clinical workflow to react to alarms generated by the system.

In summary, we have developed a comprehensive monitoring system for the entire respiratory failure management process. We have shown that our system has the potential to facilitate early identification and assessment of deteriorating patients, aiming to enable rapid treatment; and to simplify resource planning within the ICU environment. The physiological relationship between risks and individual predictions can be inspected using SHAP values, thereby hopefully offering valuable insights to clinicians, and ultimately increasing trust in the system^72^. The potential benefit of the system in improving patient outcomes needs to be validated in prospective clinical implementation trials.

## Methods

### Study design and setting

The study was designed as a retrospective cohort study to develop and validate a set of clinical prediction models that are combined to form an ML-based RMS. The study was performed using data from the Department of Intensive Care Medicine at the University Hospital Bern, an interdisciplinary unit admitting >6500 patients per year and the sole provider of intensive care for adults at this hospital. This dataset (HiRID-II) was used for model development and internal validation. For external validation, an open-source dataset from the Amsterdam University Medical Center, referred to as UMCdb^21^, was used and harmonized to match the same structure of the HiRID-II dataset.

The competent ethics committee (CEC) of the Canton of Bern approved the study (BASEC 2016 01463). The need for obtaining informed patient consent for patient data from the University Hospital of Bern was waived due to the retrospective and observational nature of the study and the use of anonymized data only. No IRB or CEC approval is required for the anonymous public external validation dataset from Amsterdam University Medical Center (BASEC Req-2024-00250).

Details about participants and patient inclusion criteria in the two datasets are described in Table 1/Supplementary Fig. 1.

### HiRID-II data

For this work, we prepared the second version of the High time Resolution Intensive Care Unit Dataset (HiRID-II), consisting of high-temporal-resolution data from over 55,000 patient admissions to the intensive care units (ICUs) at the Bern University Hospital in Switzerland between January 2008 and June 2019.

HiRID-II is an improvement and update of the first HiRID dataset released by Faltys et al. on Physionet^73^, which contains over 33,000 patient admissions dating between January 2008 and August 2016. HiRID-II additionally includes patients without data for determining circulatory failure or receiving any form of full mechanical circulatory support (previously excluded from HiRID-I) and patient data between August 2016 and June 2019. The final dataset was obtained after applying exclusion criteria to 74,142 initial admissions (see flow chart in Supplementary Fig. 1).

HiRID-I includes 681 variables recorded in the patient data management system (PDMS, GE Centricity Critical Care, General Electrics), which were merged into 209 meta-variables based on their clinical concepts. HiRID-II records 218 variables, more than the HiRID-I dataset, and contains 113 more meta-variables after variable merging. It is planned to release a version of HiRID-II on Physionet that includes the new admissions and variables. Details about meta-variables are listed in Supplementary Data 1.

### Anonymization procedure

To ensure the anonymization of individuals in the dataset, we followed the same procedures that were applied to the MIMIC-III and AmsterdamUMCdb datasets; which adopted the Health Insurance Portability and Accountability Act (HIPAA) Safe Harbor requirements and, in the case of AmsterdamUMCdb, also the European Union’s General Data Protection Regulation (GDPR) standards^74,75^.

This included the removal of all eighteen identifying data elements listed in HIPAA. Free text was removed from the dataset. Patient age, height and weight were grouped into bins of size 5, with patients aged 90 years and older binned together. K-anonymization was subsequently applied to the patient’s age, weight, height, and sex. This procedure was separately applied to the original HiRID-I dataset (anonymized by Faltys et al.), including the additional set of training patients (2008–2018), and the held-out test set (2018–2019).

Within these temporally distinct training and test sets, admission dates were shifted by a random offset to lie between 2100 and 2200, while preserving seasonality, time of day, and day of week. Measurements and medications with changing units over time were standardized to the latest unit used, to ensure that the admission time point could not be deduced from the units used.

### Data splits

The publicly-released temporal data split into development and test set was used as a basis for designing the data splits in HiRID-II; after implementing the K-anonymization procedure described above. The test set of this split was held out and not used prior to generating the final results to avoid subtle overfitting during the model design process. The development set was further divided randomly by complete patients, allocating 80% of the patients to the final training set, and 20% of the patients to the validation set. The final training set was used for model training. The validation set was used for selecting optimal hyperparameters, as well as early stopping of the training process. Performance in the validation set guided model design decisions in the prototyping phase as well as the selection of clinical parameters using greedy forward selection. This splitting procedure into training and validation sets was repeated independently 5 times, to produce 5 splits.

The model development dataset drawn from HiRID-II contains 51,457 patients and the test set 4401 patients. A temporal splitting strategy analogous to the one presented by Hyland et al.^7^ was used, with one fixed test set to minimize leakage of admission time information. Five independent partitions into the training and validation sets were extracted from the development dataset to estimate variation of model performance, containing 41,165 and 10,292 patients, respectively.

For UMCdb a fixed test set consisting of a 25% random sample of patients was drawn. The remaining patients formed the development dataset, which was partitioned by five random splits in 80:20 proportion into training and validation sets. All five training/validation splits shared the same above-mentioned test set.

### Analysis platform

After extraction of the HiRID-II and UMCdb data all computational analyses were performed on a secure compute cluster environment located at ETH Zürich (https://scicomp.ethz.ch/wiki/Leonhard). Python3, with numpy, pandas, matplotlib and scikit-learn, formed the backbone of the data-processing pipeline. The LightGBM^27^ package was used for model training. Processing was performed in a batched form across most steps of the processing pipeline, with the HiRID-II dataset being split into 100 batches, and the UMCdb dataset being split into 50 batches.

### Data preprocessing, variable merging, artifact rejection, medication preprocessing

Similar to the preprocessing steps presented by Hyland et al.^7^, we first removed different types of artifacts in the data, such as timestamp artifacts and variable-misnaming artifacts, out-of-range-value artifacts, and record duplication artifacts. For variables that encode cumulative values, we converted the cumulative values to a rate. The dose values of medication variables were either converted to a rate or a binary indicator depending on their clinical relevance to respiratory failure defined by the clinicians. Drugs that are given in the form of discrete boluses were converted to continuous rates over a defined duration of action. The duration of action differs for different drugs and the details can be found in Supplementary Data 1.

After artifact removal and converting relevant variables to rates, we merged variables with the identical or near-identical clinical meaning/function into one single meta-variable (i.e., core body temperature, rectal temperature, and axillary temperature were merged to form the meta-variable temperature). Similarly, drugs with identical or equivalent compounds were merged into one medication group. For each meta-variable, we took the median value of the available measurements at each time point when any of the corresponding variables is measured.

### Data imputation

A time grid with step size of 5-min was used; with the admission time period defined as the time between the first and last heart rate measurement of the intensive care unit stay. The analyzed admission time was limited to the first 28 days of an individual ICU stay. ICU stays of more than 28 days are rare and using such data would potentially introduce a bias in the model development process.

At each grid point a binary measurement indicator column was introduced as 1 if there was an observation in the corresponding 5 min, and 0 otherwise. Further, a time to last measurement column was introduced as −1 if there is no previous observation prior to the grid point, or equal to the number of minutes since the last observation.

“Dense imputation” where every value on the grid is imputed with a finite value was used as pre-processing for endpoint annotation and label definition. In contrast, prior to feature extraction, data were gridded but only partially imputed to preserve some missingness patterns in the data. Here, a missing indicator (NAN) was left at grid points where the value could not be estimated in a clinically plausible way. Subsequently, we refer to the latter mode as “feature imputation”.

For each meta-variable in the HiRID-II data schema, an imputation algorithm was defined and applied. If there was no prior measurement before a grid point, the grid point was filled with a missing-value indicator (for “feature imputation”) or a clinically defined normal value (for “dense imputation”). If there was more than one observation with the same time-stamp, the mean of all such observations was used. The imputation modes were:

- Indefinite forward filling: The last measurement was indefinitely forward filled to each later time point on the 5-min grid.
- Limited forward filling based on medical concepts: Each measurement was forward filled up to a maximum time of *k* minutes, with *k* manually specified based on clinical concepts.
- Limited data-adaptive forward filling: The median and standard deviation of the observation intervals for a clinical parameter were estimated in the training set. Using these estimates, forward filling was applied for up to 2 × median(interval) + IQR(interval). This method was used if the forward filling horizon cannot be specified based on medical concepts.
- Attribute to exact grid point: No forward filling was used if a measurement is only relevant for a very short time at the exact grid point where it was observed. In this case, forward filling was limited to 5 min, i.e., only the grid point next to the measurement location contains the value.

The imputation modes used for each variable are listed in Supplementary Data 2, per clinical parameter.

For the variables “Cardiac output”, “Urine output”, “Fluid input”, “Fluid output”, imputation used special formulae to estimate the current value based on the patient’s observed/estimated height/weight and BMI values. More details are given in Supplementary Data 2. For static variables, analogously to time series variables, “dense imputation” guaranteed there are no missing values, and median/mode imputation based on statistics from the training set was used for continuous variables and categorical variables, respectively. “Feature imputation” for static variables used no imputation, missing values were left as NAN.

### PaO_2_ estimation

The annotation of respiratory failure depends on the availability of a current PaO2 value. To measure PaO_2_, an arterial blood gas sample (ABGA) of the patient has to be drawn and processed. Therefore, PaO_2_ measurements are only available at intervals determined by ABGA measurement frequency. For a continuous assessment of a patient’s respiratory state using P/F ratio, estimates of PaO_2_ values have to be used when measurements are not available. In the clinical setting, continuously monitored pulse oximetry-derived hemoglobin oxygen saturation (SpO_2_) can be used to estimate the current PaO_2_ value^25,76,77^. To reduce the effect of outliers, the SpO_2_ time series was pre-processed with a percentile smoother (75% percentile kernel function, 30 min centralized kernel window). A literature review of existing models revealed that the non-linear parametric model by Ellis^25,77^ performs best. We were able to further improve upon Ellis in PaO_2_ estimation using 2 nested regularized L2 regression models by using 7 hand-crafted features, defined at each time point of the stay. Polynomial features of degree 3 were then computed on these features to capture nonlinear interactions explicitly. The following features were used:

- Last available SpO_2_ measurement
- Last available PaO_2_ measurement
- Last available SaO_2_ measurement
- Last available pH measurement
- Time to last available SpO_2_ measurement
- Time to last available PaO_2_ measurement
- Closest SpO_2_ measurement to the last PaO_2_ measurement

This base model was nested into a meta-model, which performed the final prediction. As input features the meta-model used (1) the same polynomial features of the base model, (2) the prediction made by the base model as well as (3) the prior mistakes, i.e., signed offsets between base model prediction and ground-truth of the (up to) 10 prior PaO_2_ real measurements. This allows the model to adapt using the context of previous wrong predictions to improve its predictions over the time of the ICU admission, and adapt to the patient physiology at hand. Hereby the prediction of PaO_2_ is independent of the F_I_O_2_, since complex physiological interactions between F_I_O_2_ and the P/F ratio exist, which may lead to unwanted mathematical coupling between our PaO_2_ estimate and the P/F index.

The model was only trained using time points where at least one prior measurement was available for each of SpO_2_, PaO_2_, SaO_2_, and pH and a PaO_2_ measurement was recorded at this time point. The regression label was equal to the ground-truth PaO_2_ measurement. During evaluation, a prediction was only made if at least one PaO_2_ measurement was previously observed. Before the first PaO_2_ measurement, the normal imputation algorithm for PaO_2_ (forward filling) was used instead of the prediction model.

For both base model and meta model, an L2 regression loss function with a Huber regularizer was used, and using a separate validation set, the regularization weight alpha was optimized over the range [1.0,0.1,0.01,0.001,0.0001]. In the loss function the samples were weighted according to the formula 10 + 100/(1 + exp(0.025 × (RealPaO_2_-110))), to give true PaO_2_ values close to the relevant decision boundaries for respiratory failure annotation higher weights. Model development of the PaO_2_ estimation model did not use the held-out test set, which was not used prior to the final result preparation. The parameters of the PaO_2_ estimation model and the hyperparameter search grids are listed in Supplementary Table 8.

### F_I_O_2_ estimation

For the calculation of the P/F ratio, estimates of F_I_O_2_ values were necessary for every grid point. Three situations need to be distinguished: (1) the patient is breathing ambient air, i.e., F_I_O_2_ = 21% (the ambient air oxygen fraction); (2) the patient is receiving supplemental oxygen and the corresponding F_I_O_2_ is recorded in the data; (3) for patients on mechanical ventilation F_I_O_2_ is controlled by the ventilator and its value is recorded in the data. F_I_O_2_ estimation at every grid point is implemented in the following way.

- F_I_O_2_ is forward filled from the last F_I_O_2_ measurement, if (1) it was within the last 30 min, and (2) the patient was estimated to be mechanically ventilated (using the ventilation detection algorithm described later) or the ventilation mode is NIV (non-invasive ventilation).
- Otherwise, the two supplementary oxygen variables (supplemental F_I_O_2_ [%] and high flow F_I_O_2_ [%]) were considered, if a measurement was available in the last 12 h. Hereby, supplemental F_I_O_2_ [%] takes precedence if it was available in the last 12 h.
- If there was no measurement in the two supplementary oxygen F_I_O_2_ variables in the last 12 h, then an ambient air assumption was made, and F_I_O_2_ was estimated as 21%.

### Estimation of the P/F index

The P/F index (or ratio) at each grid point was defined as PaO_2_ estimate/F_I_O_2_ estimate, where the PaO_2_, F_I_O_2_ estimates at the grid point were found by the two schemas explained above. As post-processing, a Nadaraya Watson kernel smoother with a bandwidth of 20 was applied to the tentative P/F indices, to yield the final estimated P/F ratios per grid time point.

### Respiratory failure annotation

Lung function is clinically evaluated using the ratio of blood oxygen partial pressure (PaO_2_) and fraction of inspired oxygen (F_I_O_2_) as an indicator of venous admixture, commonly referred to as P/F ratio^78^. A healthy person breathing room air is expected to have a P/F ratio of approximately 475 mmHg (PaO_2_: ∼100 mmHg, room air F_I_O_2_: 21%). Current medical literature defines acute respiratory failure in three stages^79^:

- **Mild:** 200 mmHg ≤ P/F ratio <300 mmHg
- **Moderate:** 100 mmHg ≤ P/F ratio <200 mmHg
- **Severe:** P/F ratio < 100 mmHg

A grid point was labeled with the 3 severity levels or “stable” using a forward facing window of length 1 h. If two thirds of the grid points satisfied the severe criterion (i.e., <100 mmHg), it was labeled as “severe respiratory failure”, otherwise if two thirds of the grid points satisfied the moderate criterion (i.e., <200 mmHg), it was labeled as “moderate respiratory failure”, otherwise if two thirds of the grid points satisfied the mild criterion (i.e., <300 mmHg), it was labeled as “mild respiratory failure”. Otherwise the patient was labeled as “stable” at the grid point. If for at least two thirds of the grid points, the P/F ratio could not be estimated, the respiratory failure status of the grid point was set to “Unknown”. In addition to satisfying the condition on the P/F ratio in the window, we also required that the patient was in a consistent ventilation state during the grid-points where the P/F ratio criterion was satisfied (patient is not ventilated, or patient is ventilated and PEEP is not densely available, or patient is ventilated and PEEP is densely available and satisfying PEEP ≥ 4).

Because the labeling algorithm with a forward facing window can mis-label points on the right boundary of events as “not in failure”, the right edges of events were manually corrected by scanning right-wards from the tentative right edge of the event and setting the grid point to the respective severity level if the current P/F ratio actually satisfied the criterion, but was mis-labeled as not satisfying the criterion due to the forward-facing 1 h window.

As a last step, a post processing was performed where small events (length no longer than 4 h) that are sandwiched between two other events, (1) at least one of which was longer than the sandwiched event, and (2) the 2 surrounding events had the same severity label, were relabeled to match the label of the surrounding events. In this way, spuriously labeled short respiratory failure events shorter than 4 h were deleted. Moreover, small gaps between two respiratory failure events were deleted and the two events merged together.

### Sensitivity analysis with alternative respiratory failure annotations

A sensitivity analysis with the following alternative methods of annotating respiratory failure was conducted.

- Defining respiratory failure by a P/F ratio threshold of 150 mmHg instead of 200 mmHg as in the main results.
- Using a simpler PaO_2_ estimation model (Severinghaus–Ellis baseline^25,76^) instead of the ML-based imputation model.
- Defining respiratory failure directly by the S/F ratio, using an equivalent S/F ratio cutoff of 235^80^, instead of using a P/F ratio threshold of 200 mmHg.

### Ventilation status annotation

To derive the ventilation status (binary) at each grid point, a voting algorithm was used, which was informed by prior medical knowledge. Each criterion was evaluated per grid point, and depending on the outcome, positive or negative points were assigned. Positive points correspond to a higher likelihood of ventilation at a grid point, negative points to a lower likelihood of ventilation. Finally, a cut-off on the total sum was specified, using prior medical knowledge, and by judging the correctness of endpoints visually using a time series visualization toolkit, developed for this project. Points assigned by the voting system were as follows:

- +1 point: if patient was admitted before 2009/12/06, to take into account different recording of ventilation information in the PDMS before this data
- +2 points: if in a centered 30 min window on the grid point, at least one EtCO_2_ measurement of >0.5 mmHg was observed, indicating active invasive or non-invasive mechanical ventilation
- +1 point: if the current estimated ventilation mode is 2 (controlled mode) or 3 (spontaneous mode)
−1 point: if the current estimated ventilation mode is 1 (standby)
−2 points: if the current estimated ventilation mode is 4 (NIV), 5 (High flow) or 6 (CPAP)
- +1 point: if the estimated tidal volume (TV) is >0 mL
- +2 points: if the tracheotomy indicator (vm313) or intubation indicator (vm312) or Airway category (vm66) is “Intubated” or “Tracheostomy”
−1 point: No airway, Airway category (vm66) is “Maske” (mask), “Helm” (helmet), “Mundstueck” (mouthpiece), “Nasenmaske” (nasal interface).

If the combined score is at least 4 at a grid point, the ventilation status is “True”, “False” otherwise.

Thereafter, post-processing was applied:

1. We removed gaps which were likely caused by the patient leaving the ICU for procedures. The gaps were detected by missing heart rate information during the period in question (fewer than 50% of the time points in the gap had at least one additional HR observation within 10 min). The patient was assumed to be ventilated during these gaps. Gaps shorter than 15 min between successive ventilation episodes were removed and uninterrupted ventilation was assumed.
2. Gaps shorter than 24 h were closed in case a patient had a tracheotomy indicator before and after the event (normal tracheostomy weaning procedure).
3. Short ventilation events of length shorter than 45 min were deleted, if they did not occur at the beginning of the stay (i.e., no heart rate was recorded before the event), as these are likely spurious detections.

### Readiness to extubate annotation

Readiness to extubate status was only annotated for time points where the patient was mechanically ventilated according to the criteria mentioned above. It was informed by medical prior knowledge and at each time point the number of violations of commonly accepted extubation criteria was counted, to form a scoring system:

- Ventilator mode is not 3 (spontaneous breathing), the patient cannot be extubated. A violation score of +9 is assigned. For data from prior to 2010 this criterion was not applied as the ventilator mode was sometimes incorrectly recorded in the data.
- Current PEEP is >7: violation score of +3
- Current pressure support is >10: violation score of +3
- Current F_I_O_2_ is >0.4: violation score of +3
- The rapid shallow breathing index (1000 × RR/TV) is at least 105: violation score of +3
- Current RR is at least 35 breaths/min: violation score of +3
- Current MV (Minute volume) is at least 10 L/min: violation score of +3
- Current P/F ratio (as estimated using the annotation algorithm for respiratory failure) is ≤150 mmHg: violation score of +3
- Current PaCO_2_ is at least 50 mmHg: violation score of +3
- Glasgow coma scale (GCS) is ≤8: violation score of +1
- Current mean arterial pressure (MAP) is ≤60 mmHg: violation score of +1
- Standardized dose of norepinephrine is >0.05/µg/kg/h or any dose of inotropes (epinephrine, dobutamine, milrinone, levosimendan, theophylline): violation score of +1
- Current lactate is ≥2.5 mmol/L: violation score of +1

If the sum of violation scores from the 13 criteria at any time-point is <9, the patient was assumed (tentative) to be ready for extubation. To increase the robustness of the annotation, a backward window of length 1 h was used, and the patient was assumed to be ready for extubation if two thirds of time-points in the last hour satisfied the criteria. The coefficients of the scoring system were obtained by fitting a model to predict extubation failure from the input variables, and then rounding the coefficients to be integers. The threshold of 9 points was determined by visually inspecting the time series annotated with integer scores to check for clinical plausibility.

### Extubation failure (EF) task

For the purpose of extubation failure prediction, decannulations from tracheostomy were not considered. An extubation was defined as a transition from ventilated to non-ventilated status, where the annotation algorithm for ventilation detection was used. The label for extubation failure was defined as positive if the patient was re-intubated within the next 48 h after the extubation event. The re-intubation was ignored if the patient was away from the ICU in the hour immediately prior to the re-intubation (detected by a HR measurement gap of two thirds of the hour), which might indicate that the re-intubation was for procedural reasons, not for respiratory failure. If a valid re-intubation occurred, the label for extubation failure was positive otherwise negative. If the patient died within the next 48 h after extubation, and no re-intubation occurred, the label was treated as uncertain. Sample augmentation was used for training and evaluation in the near vicinity of extubations, i.e., the prior 30 min before an extubation share the same label (extubation failure or no extubation failure) as the exact time point of the extubation. In this way, the number of training samples is increased, and a clinically reasonable assumption is made that the physiological state reflecting likelihood of extubation failure does not change within a time span of 30 min.

### Respiratory failure onset (RF) task

We are interested in predicting the onset of hypoxemic respiratory failure of moderate or severe level, as previously defined. The ML label was only defined at time points where the patient was not already in respiratory failure (P/F ratio <200 mmHg) and the annotation was not “unknown”. We assigned a positive label if the patient was currently stable or in mild respiratory failure, but moderate or severe respiratory failure occurred at some point in the next 24 h. The label was undefined if the respiratory status was “unknown” at the current time point or for the entire next 24 h.

### Ventilation onset (MV_Start_) task

We are interested in predicting the onset of mechanical ventilation as defined by the score-based algorithm presented earlier. The ML label was only defined at time points where the patient was not already mechanically ventilated. If the patient was currently not ventilated, but was mechanically ventilated at some point in the next 24 h, the label was positive and negative otherwise. The 30 min just before the onset of mechanical ventilation were excluded from training and evaluation, to prevent any potential leakage of information from the future. The label was undefined if the ventilation status is “unknown” for the complete next 24 h, or at the current time point.

### Readiness to extubate onset (MV_End_) task

We are interested in predicting if an intubated patient becomes newly ready to extubate as defined above. The ML label was defined at time points where the patient was mechanically ventilated and not yet ready to be extubated. If they were currently not ready to extubate, but were ready to extubate at some point within the next 24 h, the label was positive and negative otherwise. The label was undefined if the readiness to extubate status was “unknown” for the entire next 24 h, or at the current time point.

### Feature extraction

To give our model a comprehensive view of the patient state, the following feature classes were extracted from the clinical parameters available in the HiRID-II dataset.

- *Current value:* The current time grid value of the clinical parameters in the HiRID-II dataset was used directly as a feature.
- *Time since admission:* The time since admission was used as an individual feature.
- *Endpoint annotation variables:* The current estimated value of F_I_O_2_ and the current ventilation status, as computed by the scoring algorithm, were used as additional clinical variables. The current PaO_2_ estimate was not used to avoid potential leakage of information from the future.
- *Multi-resolution summaries:* Various summary functions were computed over multiple horizons, including the last 10 h, the last 26 h, the last 63 h, and the last 156 h. These 4 horizon lengths correspond to the 20/40/60/80 percentiles of the available history across all time points in the training set. From the training set the expected number of measurements within the horizon was estimated, using the median observation interval of the parameter. If the expected number of measurements in the horizon was less than 5, the horizon was not used for feature computation. For ordinal variables, median/IQR/trend were used as the 3 summary functions. The trend was defined as the slope of a regression line fitted over the values in the horizon. For binary variables, the mean was used as the only summary function. Note, for binary variables, the mean can be also interpreted as the proportion of the horizon in which a certain condition was true. For categorical variables, the mode was used as the only summary function. All 4 horizons were computed only for important variables, which were determined using a preliminary variable importance selection analysis. The important variables are listed in Supplementary Data 3. For other variables, only the shortest of the 4 horizons, for which the expected number of measurements exceeded 5, was used.
- *Measurement intensity:* The time to last real measurement was computed as a feature. If there were no such measurement, this feature was set to a very large value. The measurement density was computed over the same multi-resolution horizons as in the previous feature category. The measurement density was defined as the number of observations in the horizon divided by the horizon length.
- *Instability history:* If applicable for a variable, up to 3 severity levels were annotated using prior medical knowledge. The fraction of time spent in each severity state over the last 8 h, as well as over the entire stay up to the current time point, was extracted. This schema was used only for a subset of variables, which were among the important variables selected in a preliminary variable selection step. The severity levels and variables used for this feature class are listed in Supplementary Table 1.
- *Static variables:* Static variables are constant for all time points of the patient’s time series and are finally concatenated to the feature vector. As static variables, the patient age, APACHE patient group, gender, emergency admission status, surgical admission status, and height were used.

### Variable selection

Variable selection was performed in a 2-step process using only the development set, and not the held-out test set.

- The 20 most important variables in terms of SHAP value magnitude were pre-selected on the validation set for the 4 tasks (Respiratory failure, Extubation failure, Ventilation onset, Readiness to extubate) separately. The “SHAP importance” of a feature was defined as the mean (over the 5 temporal splits) of the mean absolute SHAP value on predictions in the validation set for that variable. The “SHAP importance” of a variable was defined as the maximum of SHAP importance over the features derived from the clinical variable. In this way 20 variables were extracted per task. The union of the variables selected for the 4 tasks formed the initial set of “important variables”, which consisted of 31 variables, and is listed in Supplementary Data 3.
- For the initial set of important variables, more complex features were computed, according to the description in the section on feature extraction above.
- For the 2 main tasks presented in the work, Respiratory failure (RMS-RF) and Extubation failure (RMS-EF), variables were greedily forward selected from the final set of complex features on 31 variables. In each step, the variable was chosen which yielded the highest time-point-based AUPRC on the validation set, among the candidate variables to be added. The output of this procedure, which was run 5 times per temporal split, was a forward trace of 31 variables, ranked by importance. The final importance of a variable was defined as the mean reciprocal rank over the 5 splits, yielding a ranked list of 31 variables. The RMS-EF model used the top 20 variables, which included both medication and non-medication variables, and the RMS-RF model used the non-medication variables among the top 20 variables, which yielded a final set of 15 variables. For ventilation onset/readiness to extubate prediction models, the union of the variables used for the respiratory failure and extubation failure models was used. The variables used by each model are listed in Supplementary Data 3.

### Model training

The generated features for the variable sets of the RMS-RF (15 variables) and RMS-EF (20 variables), RMS-MV_Start_/RMS-MV_End_ (26 variables) predictors were passed to 4 gradient-boosted decision tree ensembles implemented in LightGBM^27^, with one separate model per task. As LightGBM is robust to missing data and different feature scales, data was not imputed or standard scaled prior to training. Trees were added to the ensemble until performance did not improve for 50 epochs in the validation set, early stopping the training process. As a criterion guiding the early stopping the time-point-based AUPRC on the validation set was used. Hyperparameters were optimized on the validation set, using the time-point-based AUPRC as a criterion. Hyperparameters were fixed for RMS-RF/RMS-MV_Start_/RMS-MV_End_, as experiments showed that early stopping was enough to find a configuration close to optimal. For RMS-EF, which had a small number of training set samples, a hyperparameter grid with 20 points was used to select the optimal model. The parameters of the LightGBM model and the hyperparameter selection grid for RMS-EF are listed in Supplementary Table 6, and those for the decision-tree baseline of RMS-RF are listed in Supplementary Table 7. Prediction scores were generated for patients in the test set, using a separate LightGBM model for each of the 4 tasks. To allow more flexible evaluation, for resource planning and joint task analysis using t-SNE, predictions were generated at all time-points of test set patients, even when the ground-truth label was undefined, i.e., for RMS-RF, while the patients were already experiencing respiratory failure.

### Resource planning

We developed a resource planning model for mechanical ventilation that predicts how many patients will require mechanical ventilation within certain time windows in the short-term future. This includes the start of mechanical ventilation for patients who are already admitted to ICU but not yet ventilated and additional non-elective patient admissions requiring mechanical ventilation. As the original dates were removed during anonymization for HIRID-II, we used an additionally provided dataset with the admission dates of the ICU patients in order to reconstruct the number of patients within the ICU and the ventilator resource use. To predict the number of ICU patients who will require ventilation in the near future, we used the outputs from the ML models trained for predicting the four respiratory system related tasks (respiratory failure, mechanical ventilation need, readiness to extubate and the extubation failure) for individual patients, as well as ICU-level information as features for the LightGBM model. The ICU-level information included the hour of the day, weekday, and the number of patients who were admitted and required mechanical ventilation in the past hour. To predict newly admitted emergency patients who will require mechanical ventilation, we trained a LightGBM model using only the ICU-level information. The parameters of the LightGBM model and the hyperparameter search grid for mechanical ventilation resource planning are listed in Supplementary Table 9.

### Model calibration

For the evaluation of model calibration, the prediction scores of time-points in the test set where the label was defined were gathered. The scores were binned between the minimum and maximum prediction scores observed in the test set, using a bin size of 0.05. The actual observed risk (proportion of true labels for time points in the bin) was then computed per bin and plotted against the bin location. As an evaluation metric of calibration the Brier score^81^ was used. Models showed sufficient calibration using the raw scores, so re-calibration using isotonic regression was not needed.

### Detectable event prevalence

In this manuscript, for predictors evaluated using event-based evaluation (RMS-RF, RMS-MV_Start_, RMS-MV_End_), “prevalence” refers to the detectable event prevalence, which represents the proportion of time during which events are expected to be detected in the ICU. The detectable event prevalence is dependent on the selected prediction horizon. The detectable event prevalence is calculated as the area under the event-based precision-recall curve of a random classifier.

### Prevalence correction for external validation

As the detectable event prevalence was different between the HiRID-II and the UMCdb test sets, we corrected the precision-recall curves for the performance on the test set of UMCdb such that the corrected prevalence matches with that in the HiRID-II test set by downscaling the false alarm number using the scaling factor *s* = (1/prev(HiRID-II)-1)/(1/prev(UMCdb)-1), as used by Hyland et al.^7^.

### Extubation-based evaluation (extubation failure)

Extubation failure was assessed using recall (percentage of extubation failures which were correctly predicted), and precision (percentage of extubation failure predictions which are correct, i.e., re-intubation occurs in the next 48 h), yielding a precision-recall curve, as well as recall/false positive rate, which defined the ROC curve.

### Event-based evaluation (respiratory failure)

We used the same event-based evaluation scheme used by Hyland et al.^7^, which measures the fraction of correctly predicted respiratory-failure events (recall) and the fraction of false alarms (1-precision).

### External validation + prevalence correction

To allow external validation, the most important parameters for training predictive models in HiRID-II were matched to variables in the AmsterdamUMCdb dataset^21^. To enable endpoint annotation at a similar granularity as in HiRID-II, a subset of patients with high time resolution for respiratory parameters (*n* = 6698 patients) was included. A dataset was then prepared by applying the same endpoint annotation and feature extraction pipeline as for HiRID-II. Because admission times are not exactly available in UMCdb, data were randomly divided by patient into a fixed test set containing 1674 patients and a development dataset of 5024 patients. To retrieve variation estimates of performance, the development dataset was partitioned five times into training and validation sets. For external validation, we applied the models trained on HiRID-II dataset to the UMCdb dataset, and used prevalence correction to re-scale the false positive count, as described in the section on “Prevalence correction”.

### Sub-cohort/Fairness analyses

We grouped patients by demographic characteristics (sex, age) and clinical characteristics (APACHE II/IV admission group). For binary grouping, we compared one group versus the other, while for multi-categorical grouping, we compared patients belonging to a group to all the other patients.

Due to the small number of patients composing certain cohorts of patients, we relied on bootstrapping. We created 100 bootstrap samples of the patients from the test set (i.e., we sample randomly with replacement from the patients composing the test set). We then computed the different performance metrics for each bootstrap sample and for each of our patient cohorts. Having several bootstrap samples to perform the analysis on allowed us to better understand the variability of the patients within each cohort and to present these in the “Results” section.

To compare groups of patients, we assumed that the samples of one cohort of patients and the other cohort are independent. However, we do not assume normality of the distribution. To compare the distribution of our metrics across different cohorts, we used the Mann-Whitney U test at a significance level of 0.1%. Since for each grouping we performed multiple tests, we corrected the *p*-value with a Bonferroni correction. We tested whether patients from a certain group are significantly worse off (according to fairness of prediction performance) compared to patients not belonging to this group. On the result plots, we mark groups that are significantly worse off in terms of prediction performance with a star.

For the respiratory failure task, we computed the precision at:

- 80% event-based recall for each cohort (the threshold will thus be different for each cohort)
- 90% event-based recall for each cohort (the threshold will thus be different for each cohort)

For the extubation failure task, we computed the precision at:

- 80% recall for each cohort (the threshold will thus be different for each cohort)
- 20% recall for each cohort (the threshold will thus be different for each cohort)

Finally, for both tasks, we also computed the corrected event-based AUPRC.

### Model inspection using SHAP values

SHAP values for the positive class were extracted using a “SHAP tree explainer” built for LightGBM ensembles, in the validation set and test set. In the validation set the mean absolute SHAP values of each feature were used to create an initial set of important variables for variable selection (refer to the section on “Variable selection” above). In the test set the signed SHAP values (Interpretation: Large SHAP value means the feature value contributes to an increase of the prediction score) were used to interpret the model’s prediction, i.e., by plotting them against the feature value at the time point when the prediction is made.

### Joint task analysis using t-SNE

For the joint task analysis, the test set predictions for the 4 tasks (i.e., RMS-RF, RMS-EF, RMS-MV_Start_, and RMS-MV_End_) were used. In principle, predictions were available at all time points in the patient stay for all tasks, irrespective of whether the label of the task was defined at the time point. For computing the t-SNE embedding, only the current value features of 16 clinical parameters were used, which correspond to the union of the top 10 important variables for the RMS-RF/RMS-EF models. As t-SNE requires dense input without missing values, the “dense imputation” data (refer to the section on Imputation for its definition) was used as an input for t-SNE. Prior to fitting of the t-SNE map, the data was standard scaled such that each dimension had mean 0 and standard deviation 1, such that all variables have equal importance in the t-SNE input space. A random subsample of 150,000 time points in the test set was drawn to allow fast fitting of the embedding algorithm. The t-SNE map was computed once, independent of task, as it only depends on the clinical parameters but not on the prediction scores of the 4 models. For fitting t-SNE, the implementation available in the Python package scikit-learn (https://scikit-learn.org/stable/modules/generated/sklearn.manifold.TSNE.html) was used with default parameters, and target dimension 2. In the t-SNE plots, only hexes with at least 30 assigned time points were displayed, ignoring very rarely used parts of the embedding space.

### Statistical methods

In result plots the solid curves refer to the mean of the performances obtained in the five experimental replicates, corresponding to the five temporal splits, as described in the section on data splits. Light shaded regions refer to the standard deviation of performances obtained in the five experiment replicates.

## Supplementary information

Supplementary Information

Supplementary Data 1

Supplementary Data 2

Supplementary Data 3

## Data Availability

The manuscript presents the High Time Resolution Intensive Care Unit Dataset II (HiRID-II), a substantial update to HiRID-I, that will be made available to the research community on https://github.com/ratschlab/hirid2 (previous version available here: https://physionet.org/content/hirid/1.1.1/). More information on HiRID-I is available on https://github.com/ratschlab/hirid/.

## Code availability

The code used in this research is available at https://github.com/ratschlab/RMS under an open-source license.
