# Supplementary material for: RMS: a ML-based system for ICU respiratory monitoring and resource planning
Source: NPJ Digit Med. 2025 Dec 19;8:775. doi: 10.1038/s41746-025-02081-4 (PMC12722767; doi:10.1038/s41746-025-02081-4)
Supplement: Supplementary file 1 — Supplementary Information [file 41746_2025_2081_MOESM1_ESM.pdf]

Supplementary Information

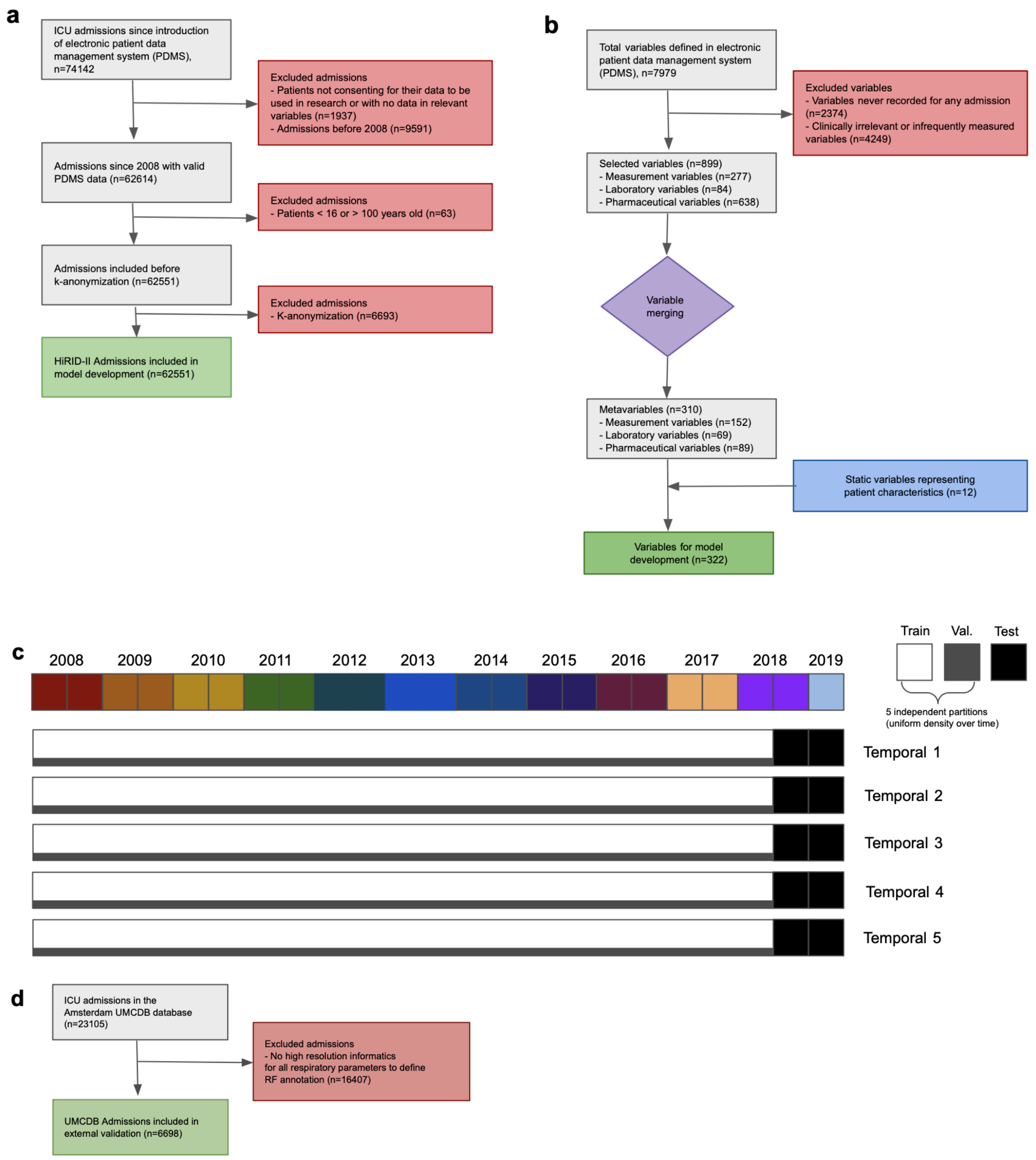

Supplementary Figure 1 | Patient inclusion & Experimental design. a. Patient inclusion schema in the HiRID-II dataset. **b.** Inclusion of clinical parameters in the data extraction pipeline of the HiRID-II dataset. **c.** Split design schema for performance evaluation. A fixed test set consisting of admissions starting in Mid June 2018 to the end of 2019 was used, which is shared by all five temporal splits, and is marked by a black block in all five splits. The remaining patients were randomly partitioned five times into a training and validation set, each defining a temporal split, which is indicated by the horizontal white and grey bars. **d.** Patient inclusion schema in the UMCdb dataset used for external validation.

a

	Respiratory failure	No respiratory failure
**Alive at discharge**	22134 (90.7 %)	35029 (97.0 %)
**Dead at discharge**	2264 (9.3 %)	1077 (3.0 %)

b

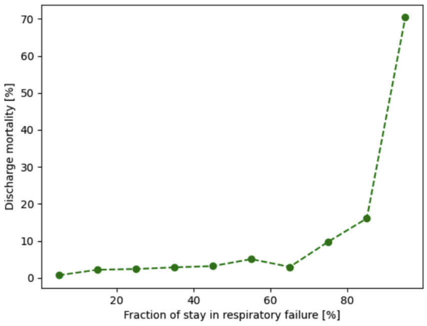

c

	Extubation failure	No extubation failure
**Alive at discharge**	2419 (92.1 %)	18716 (99.6 %)
**Dead at discharge**	208 (7.9 %)	67 (0.4 %)

d

	Mechanically ventilated	Not mechanically ventilated
**Alive at discharge**	22898 (88.8 %)	34265 (98.7 %)
**Dead at discharge**	2892 (11.2 %)	449 (1.3 %)

e

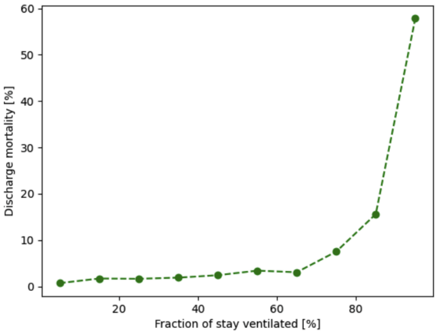

Supplementary Figure 2 | Association of ICU Mortality with Respiratory Failure, Extubation Failure, and Ventilation, respectively. a. Mortality statistics for patients with respiratory failure at some time during their ICU stay, and those without respiratory failure during their ICU stay. **b.** Relationship of ICU mortality with fraction of the ICU stay in which patients experience respiratory failure. **c.** Mortality statistics for patients with extubation failure, and those without extubation failure but with at least one successful extubation. **d.** Mortality statistics for patients receiving mechanical ventilation during their ICU stay, and those not ventilated. **e.** Relationship of ICU mortality rate with fraction of their stay during which patients are mechanically ventilated.

a

Evaluation setting	Model	Absolute error [mmHg]
All PaO_2 samples	PaO_2 estimation model	9.77 ± 0.04 [4.20 ± 0.04,21.31 ± 0.11]
All PaO_2 samples	Severinghaus-Ellis Baseline	12.9 ± 0.13 [5.49 ± 0.07,33.51 ± 0.42]
No PaO_2 in last 8h	PaO_2 estimation model	11.74 ± 0.08 [4.98 ± 0.03,27.18 ± 0.4]
No PaO_2 in last 8h	Severinghaus-Ellis Baseline	13.7 ± 0.21 [5.71 ± 0.09,37.38 ± 0.59]

b

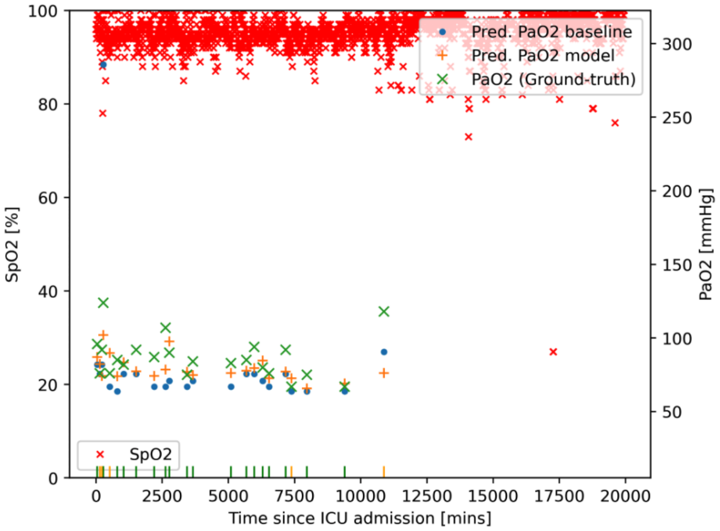

Supplementary Figure 3 | Performance of PaO_2 estimation models. a. Performance evaluation of PaO_2-estimation model on HiRID-II test set in terms of MAE vs. ground-truth PaO_2 from invasive blood tests, compared with the non-linear Severinghaus-Ellis baseline. Error bars were obtained by re-sampling the test set with 50 %, 5 times at random, using complete patients. **b.** Example time series of predicted and ground-truth PaO_2 values, as well as SpO_2 values, and baseline predictions. A patient was selected at random for which the median absolute error of both model and baseline is close (within 1 mmHg) to their population median reported in panel a. The rug plot indicates time-points for which each model performs better than the other.

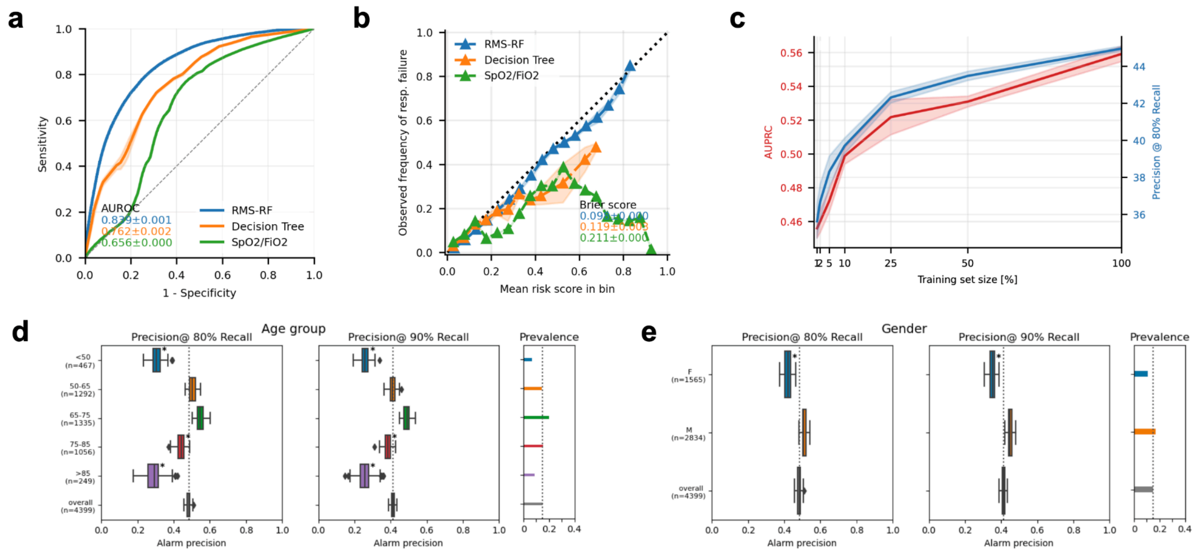

Supplementary Figure 4 | Evaluation of RMS-RF. a. ROC-based performance of the RMS-RF score, compared with the two baselines. **b.** Calibration of the RMS-RF model compared with the two baselines. **c.** Performance of the RMS-RF model, as the training set size is varied, in terms of complete patients **d.** Performance of the RMS-RF model by age group, for event recalls of 80/90 %. The model was re-calibrated for each sub-group using information available at admission time, to achieve a comparable event recall. When the prevalence of an event decreases, a greater proportion of positive results will be false, reducing the test's precision. **e.** Performance of the RMS-RF model by gender, for event recalls of 80/90 %. The model was re-calibrated for each sub-group using information available at admission time, to achieve a comparable event recall.

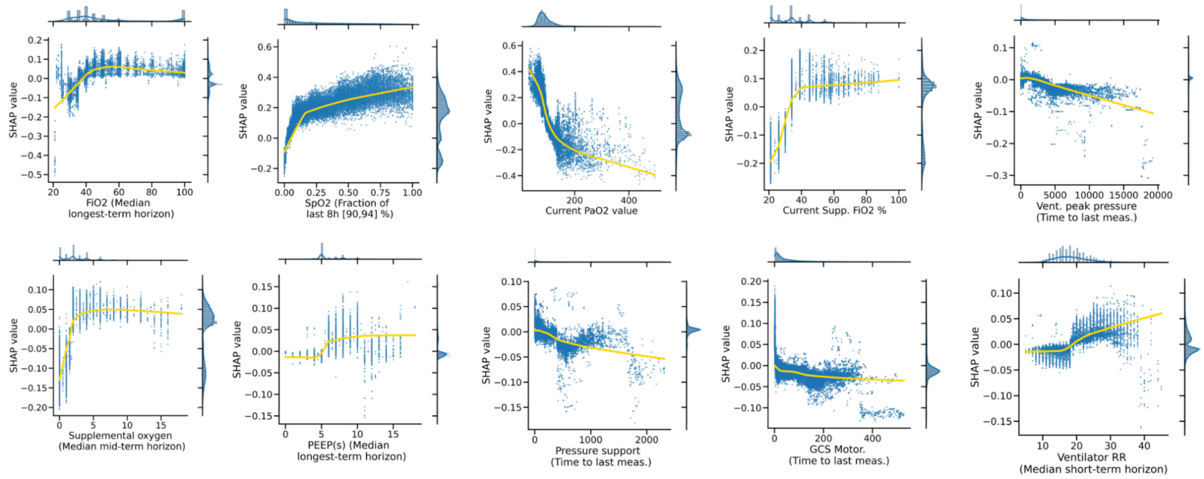

Supplementary Figure 5 | Model introspection of RMS-RF. SHAP value - feature value interactions of the top feature of the top 10 most important variables contained in RMS-RF.

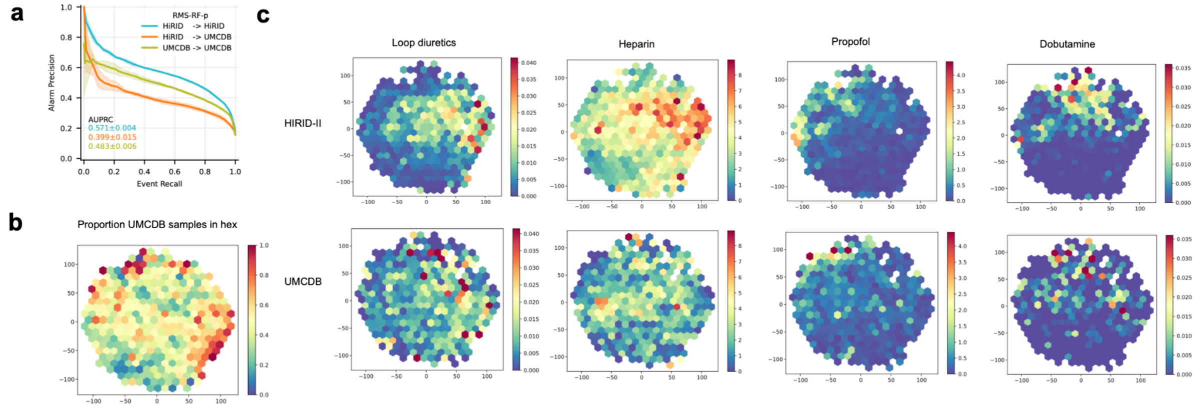

Supplementary Figure 6 | External validation of RMS-RF-p and medication policy comparison between HiRID-II and UMCdb. a. Performance of the RMS-RF-p model, which additionally includes medication variables, when trained and tested on HiRID-II, transferred to UMCdb and retrained in the UMCdb dataset **b.** t-SNE embedding of time points in the test set of a pooled dataset between samples from HiRID-II and UMCdb (1:1 ratio of two datasets), of physiological parameters. Only time points when the patient is not in respiratory failure are taken into account, for which the RMS-RF-p model is active. The color indicates the proportion of time points in the UMCdb dataset in a given hex. **c.** The same t-SNE embedding as in b is displayed separately for time points from the HiRID-II dataset, and the UMCdb dataset, corresponding to the rows. The hexes in the t-SNE are colored by the mean drug dosage of all time points assigned to the hex. The four medication variables, for which transfer issues of the RMS-RF-p model were detected, are analyzed in the columns. Medication policy differences are visible for all four variables, in particular for Heparin & Propofol.

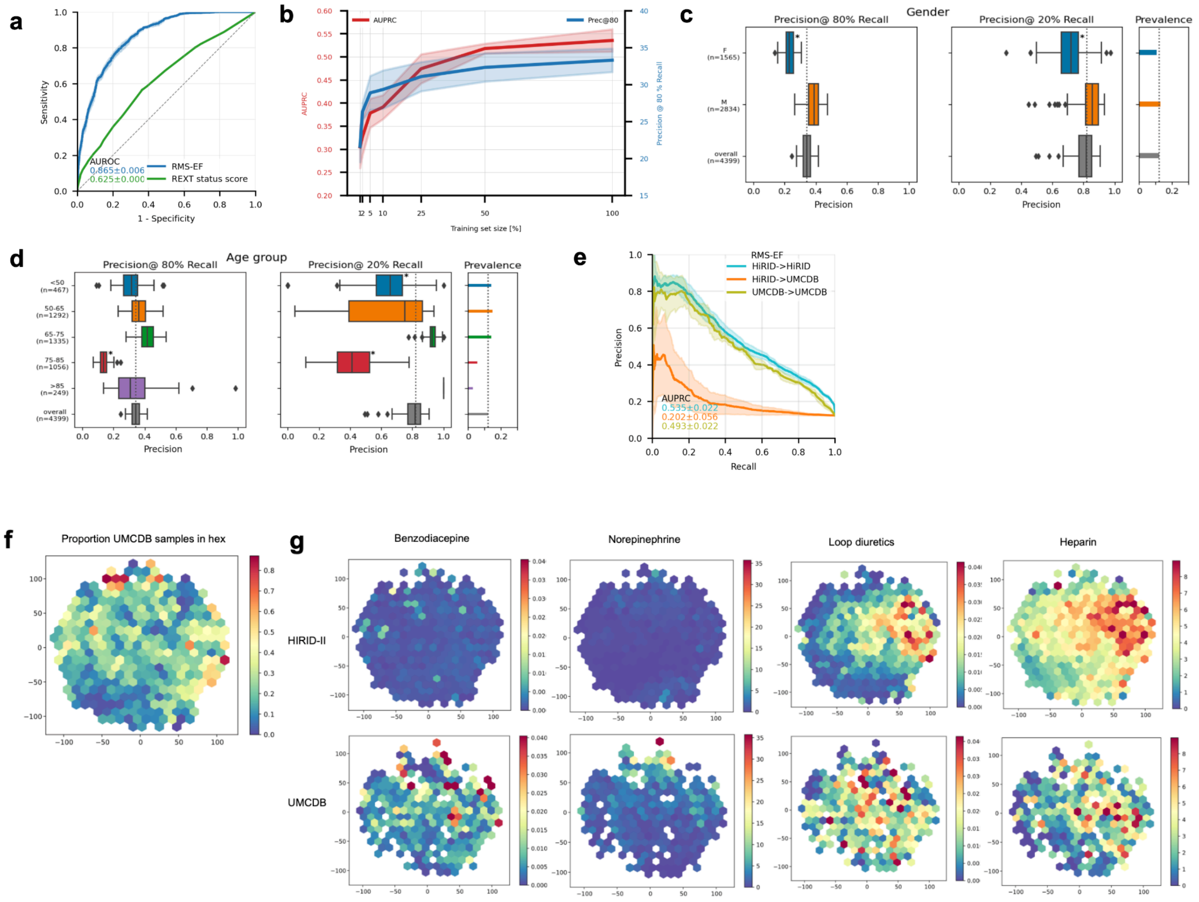

Supplementary Figure 7 | Evaluation of RMS-EF and Medication policy comparison between HiRID-II and UMCdb. a. ROC-based performance of RMS-EF, compared with the baseline. **b.** Performance of RMS-EF as the training set size is varied between 1 % and 100 % of the original dataset size, by subsampling complete patient records in the training set. **c.** Performance of RMS-EF stratified by gender, at recall of 80/20 %. The model was re-calibrated for each sub-group using information available at the time of admission, to achieve a comparable recall. **d.** Performance of RMS-EF for different age groups, at recalls of 80/20 %. The model was re-calibrated for each sub-group using information available at the time of admission, to achieve a comparable recall. **e.** Performance of the RMS-EF model, when trained/tested in the HiRID-II dataset, transferred to the UMCdb dataset, and retrained in the UMCdb dataset. **f.** t-SNE embedding of time points in the test set of a pooled dataset between samples from HiRID-II and UMCdb (1:1 ratio of two datasets), of physiological input variables. Only time points when the patient is ready-to-extubate are taken into account, for which the RMS-EF model is active. The color indicates the proportion of time points in the UMCdb dataset in a given hex. **g.** The same t-SNE embedding as in g is displayed separately for time points from the HiRID-II dataset, and the UMCdb dataset, corresponding to the rows. The hexes in the t-SNE are colored by the mean drug dosage of all time points assigned to the hex. The four medication variables, for which transfer issues of the RMS-EF model were detected, are analyzed in the columns. Medication policy differences are visible for all four variables, in particular for Benzodiacepine & Norepinephrine.

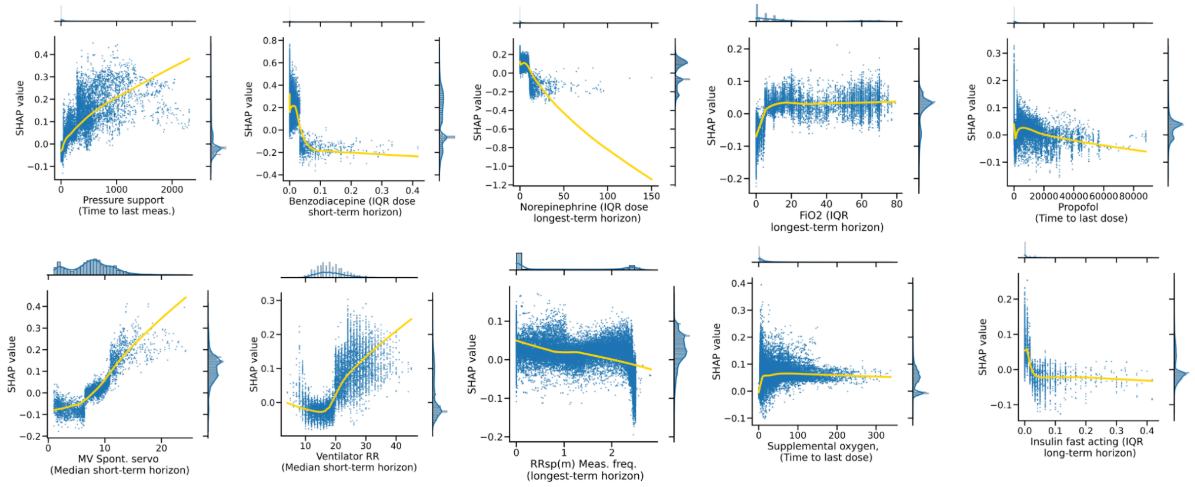

Supplementary Figure 8 | Model introspection of RMS-EF. SHAP value - feature value interactions for the top feature of the top 10 most important variables contained in the RMS-EF model.

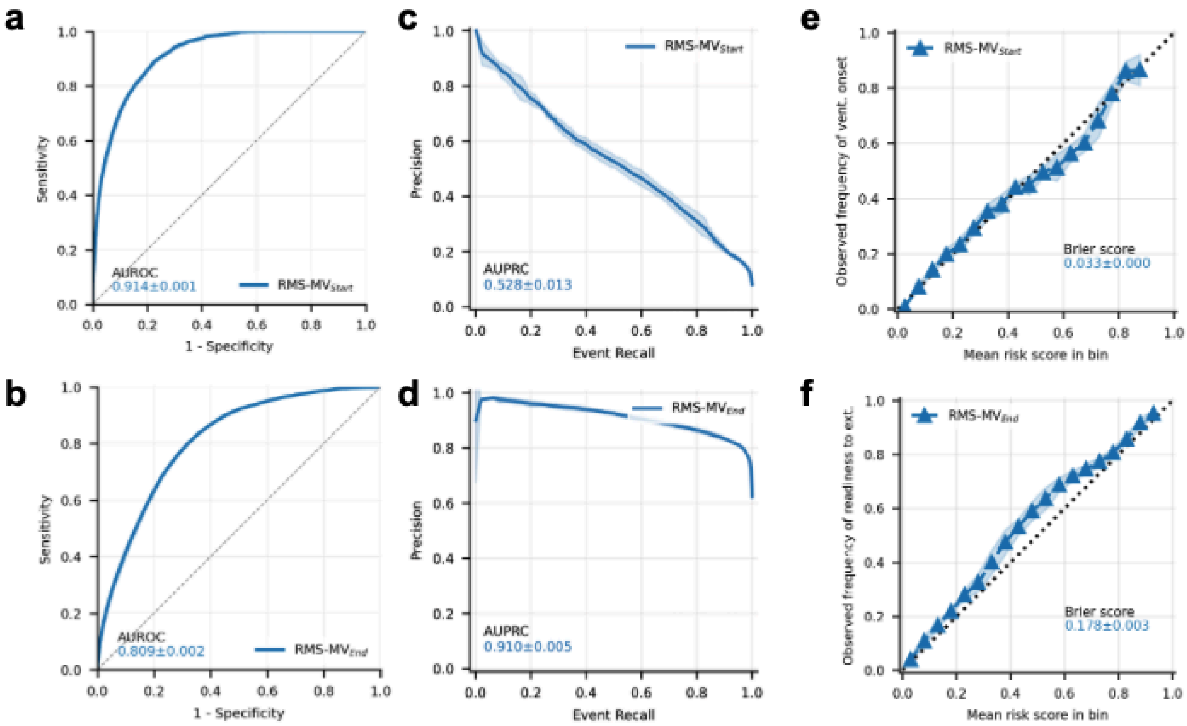

Supplementary Figure 9 | Evaluation of RMS-MV$_{Start}$/RMS-MV$_{End}$. a. ROC-based performance of RMS-MV$_{Start}$, predicting ventilation onset within the next 24h. **b.** ROC-based performance of RMS-MV$_{end}$, predicting being newly ready to extubate within the next 24h. **c.** Event-based PRC of the RMS-MV$_{Start}$ alarm system. **d.** Event-based PRC of the RMS-MV$_{End}$ alarm system. **e.** Calibration of the RMS-MV$_{Start}$ score. **f.** Calibration of the RMS-MV$_{End}$ score.

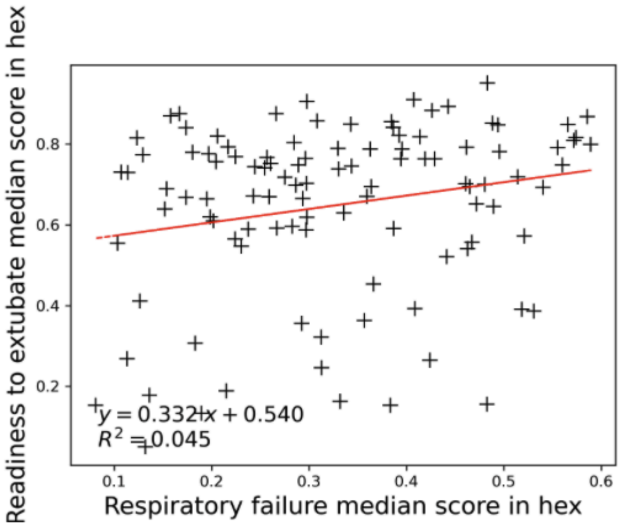

Supplementary Figure 10 | Joint task analysis details. Scatter plot of median respiratory failure vs. median readiness to extubate score in the hexes analyzed in the explorative joint analysis of RMS scores (see Fig. 5). A light positive correlation between respiratory failure and readiness to extubate scores can be observed, which is barely significant at 5 % level. A Wald test which tests non-zero slope of the regression line (shown in red) was performed.

Meta-variable ID	Variable name	Severity stage 1	Severity stage 2	Severity stage 3
vm58	FiO2	[30,40]	[40,60]	[60,100]
vm20	SpO2	[90,94]	[86,90]	[0,86]
vm23	Supplemental oxygen	[2,4]	[4,8]	[8,12]
vm141	SaO2	[90,94]	[86,90]	[0,86]
vm140	PaO2	[62,80]	[55,62]	[0,55]
vm309	Supplemental FiO2 %	[21,40]	[40,60]	[60,100]
vm62	Ventilator peak pressure	[30,40]	[40,45]	>45
vm25	GCS Response	[4,4]	[3,3]	[1,2]
vm26	GCS Motor	[5,5]	[3,4]	[1,2]
vm22	Respiratory rate	[12,20]	[20,25]	>25
fio2estimated	FiO2 estimate	[30,40]	[40,60]	[60,100]

Supplementary Table 1 | List of severity levels for computing 'instability history' features, for a subset of the important variables.

a.

Illness severity group	# Patients	Event prevalence	AUROC Mean (Std)	Prev-corrected AUPRC Mean (Std)
Max. APACHE score <20	1962 (49.0 %)	0.074	0.83 (0.002)	0.461 (0.003)
Max. APACHE score >=20, <25	738 (18.4 %)	0.124	0.79 (0.003)	0.520 (0.006)
Max. APACHE score >=25, <30	660 (16.5 %)	0.215	0.80 (0.004)	0.463 (0.005)
Max. APACHE score >=30, <35	339 (8.5 %)	0.306	0.78 (0.001)	0.426 (0.007)
Max APACHE score >=35	308 (7.7 %)	0.368	0.74 (0.002)	0.362 (0.010)

b.

Illness severity group	# Patients	Label prevalence	AUROC Mean (Std)	Prev-corrected AUPRC Mean(Std)
Max. APACHE score <20	1962 (49.0 %)	0.039	0.88 (0.02)	0.58 (0.08)
Max. APACHE score >=20, <25	738 (18.4 %)	0.045	0.91 (0.02)	0.59 (0.06)
Max. APACHE score >=25, <30	660 (16.5 %)	0.094	0.87 (0.005)	0.55 (0.02)
Max. APACHE score >=30, <35	339 (8.5 %)	0.17	0.81 (0.03)	0.38 (0.06)
Max APACHE score >=35	308 (7.7 %)	0.3	0.79 (0.01)	0.42 (0.04)

Supplementary Table 2 | Performance of RMS-RF / RMS-EF predictors in sub-cohorts stratified by illness severity (APACHE score). a: Performance of RMS-RF. **b:** Performance of RMS-EF.

P/F ratio cutoff to define RF	Prediction horizon	Detectable event prevalence	AUROC Mean (Std)	AUPRC Mean (Std)	Prev-corrected AUPRC Mean (Std)
200 mmHg	8h	0.081	0.840 (0.001)	0.331 (0.006)	0.505 (0.006)
**200 mmHg***	**24h***	0.161	0.839 (0.001)	0.559 (0.005)	0.559 (0.005)
150 mmHg	8h	0.042	0.873 (0.001)	0.245 (0.004)	0.567 (0.003)
150 mmHg	24h	0.095	0.866 (0.001)	0.455 (0.005)	0.594 (0.004)
100 mmHg	8h	0.012	0.906 (0.002)	0.078 (0.005)	0.550 (0.012)
100 mmHg	24h	0.029	0.882 (0.004)	0.164 (0.018)	0.533 (0.027)

Supplementary Table 3 | Comparison of different P/F cutoffs for RF definition. Performance of the RMS-RF predictor in the test set, when the cutoff to define respiratory failure (P/F ratio) and the prediction horizon used to define machine learning labels for training, is varied. The detectable event prevalence indicates the percentage of time when events should be detected in the ICU , which varies based on the chosen prediction horizon.

*A P/F ratio cutoff of 200 mmHg with a 24-hour prediction horizon is used in the definition of RF in the paper.

PaO2 estimation model	Detectable event prevalence	AUROC Mean (Std)	AUPRC Mean (Std)	Prev-corrected AUPRC Mean (Std)
**LightGBM-based ML model***	0.161	0.839 (0.001)	0.559 (0.005)	0.559 (0.005)
Severinghaus-Ellis Baseline	0.201	0.847 (0.0004)	0.596 (0.003)	0.532 (0.003)

Supplementary Table 4 | Comparison of different PaO2 estimation models. Performance of the RMS-RF predictor in the test set, when the model to estimate PaO2 values from other variables, such as SpO2 is measured, is varied. We contrast the ML based model and a simpler Ellis-based baseline. A P/F ratio cutoff of 200 mmHg is used to define respiratory failure, and a prediction horizon of 24 hours for generating machine learning labels.* The LightGBM-based ML model is used for PaO2 estimation in the paper.

Cutoff to define RF	Equivalent cutoff	Detectable event prevalence	AUROC Mean (Std)	AUPRC Mean (Std)	Prev-corrected AUPRC Mean (Std)
**P/F ratio < 200 mmHg***	S/F ratio: 235	0.161	0.839 (0.001)	0.559 (0.005)	0.559 (0.005)
S/F ratio < 235 & SpO2 < 0.97	P/F ratio: 200 mmHg	0.191	0.872 (0.0003)	0.589 (0.002)	0.540 (0.002)

Supplementary Table 5 | Comparison of RF definitions based on P/F ratio and S/F ratio. Performance of the RMS-RF predictor in the test set, when the method to define respiratory failure is varied, either by using estimated PaO2 values and the P/F ratio, or an alternative approach that uses the S/F ratio directly with an equivalent cutoff as reported by Rice et al. in “Comparison of the SpO2/FIO2 ratio and the PaO2/FIO2 ratio in patients with acute lung injury or ARDS” (Chest, 2007). A prediction horizon of 24 hours was used for generating machine learning labels.

Parameter	Value	Notes
Model	LGBMClassifier	
n_estimators	5000	
random_state	2021	
num_leaves	32	{8,16,32,64,128} search space for RMS-EF
learning_rate	0.1	0.05 for RMS-EF
colsample_bytree	0.5	{0.33,0.66} search space for RMS-EF
class_weight	None	
is_unbalance	FALSE	
metric	custom	
objective	binary	
subsample_freq	1	
subsample	0.5	{0.33,0.66} search space for RMS-EF
max_depth	int(log2(num_leaves))	
subsample_for_bin	1000000	
min_child_samples	1000	50 for RMS-EF
max_cat_to_onehot	100	
cat_smooth	0	
cat_l2	0	
early_stopping_rounds	20	10 for variable selection
custom_eval_metric	AUPRC	

Supplementary Table 6 | Model training parameters and grid used for selection of hyperparameters for RMS-EF.

Parameter	Value
Model	LGBMClassifier
n_estimators	1
random_state	2021
num_leaves	32
learning_rate	0.05
colsample_bytree	1
class_weight	None
is_unbalance	FALSE
objective	binary
subsample_freq	0
subsample	1
max_depth	int(log2(num_leaves))
subsample_for_bin	1000000
min_child_samples	1000
max_cat_to_onehot	100
cat_smooth	0
cat_l2	0

Supplementary Table 7 | Model training parameters and grid used for selection of hyperparameters for the decision tree baseline to compare RMS-RF performance with.

Parameter	Value
Model	SGDRegressor
alpha	{1.0,0.1,0.01,0.001,0.0001} search space
random_state	2021
penalty	l2
loss	huber
learning_rate	adaptive
early_stopping	FALSE
sample_weights	Formula according to methods document

Supplementary Table 8 | Model training parameters and grid used for selection of hyperparameters for the PaO_2 estimation model.

Parameter	Search grid
Model	LGBMRegressor
objective	regression_l2
number of leaves	[4.0, 8.0, 16.0, 32.0, 64.0, 128.0]
number of rounds	[100.0]
stopping rounds	[10.0, 20.0]

Supplementary Table 9 | Model training parameters and grid used for selection of hyperparameters for the mechanical ventilation resource planning model.

Supplementary Data Files:

Supplementary Data File 1. Details on the clinical parameters extracted in the HiRID-II dataset (downloadable XLSX file).

Supplementary Data File 2. Details on the imputation parameters, such as normal value, and imputation models, for the clinical parameters (downloadable XLSX file).

Supplementary Data File 3. List of important variables used for computing complex features, as a basis for variable selection, and for building the final models RMS-RF/RMS-EF/RMS-MV_{Start}/RMS-MV_{End} (downloadable XLSX file).
